# Metallaelectro-catalyzed alkyne annulations *via* C–H activations for sustainable heterocycle syntheses

**DOI:** 10.1039/d4cc03871a

**Published:** 2024-09-25

**Authors:** Preeti Kushwaha, Anjali Saxena, Tristan von Münchow, Suman Dana, Biswajit Saha, Lutz Ackermann

**Affiliations:** a Amity Institute of Click chemistry Research & Studies, Amity University Noida 201303 Uttar Pradesh India; b Amity Institute of Biotechnology, Amity University Noida 201303 Uttar Pradesh India bsaha1@amity.edu; c Wöhler Research Institute for Sustainable Chemistry (WISCh), Georg-August-Universität Göttingen 37077 Göttingen Germany Lutz.Ackermann@chemie.uni-goettingen.de

## Abstract

Alkyne annulation represents a versatile and powerful strategy for the assembly of structurally complex compounds. Recent advances successfully enabled electrocatalytic alkyne annulations, significantly expanding the potential applications of this promising technique towards sustainable synthesis. The metallaelectro-catalyzed C–H activation/annulation stands out as a highly efficient approach that leverages electricity, combining the benefits of electrosynthesis with the power of transition-metal catalyzed C–H activation. Particularly attractive is the pairing of the electro-oxidative C–H activation with the valuable hydrogen evolution reaction (HER), thereby addressing the growing demand for green energy solutions. Herein, we provide an overview of the evolution of electrochemical C–H annulations with alkynes for the construction of heterocycles, with a topical focus on the underlying mechanism manifolds.

## Abstract 2

Herein, an overview of the evolution of electrochemical C–H annulations with alkynes for the construction of heterocycles is provided, with a topical focus on the underlying mechanism manifolds.

## Graphical abstract

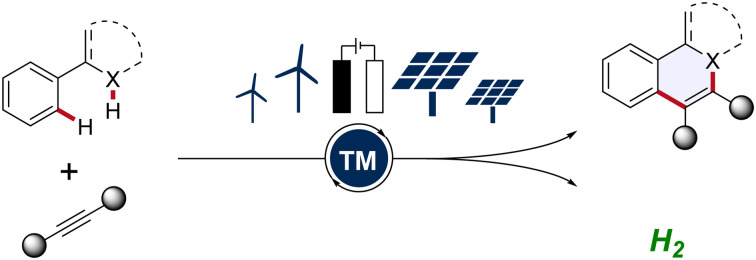

## 1 Introduction

**Fig. 1:**
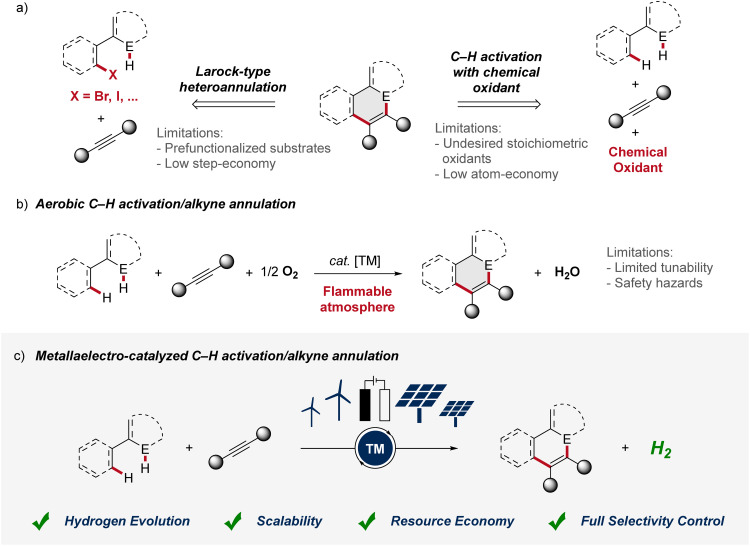
(a) Common strategies for the assembly of heterocycles *via* alkyne annulation. (b) Improved atom economy by aerobic C–H activation/annulation. (c) Metallaelectro-catalyzed C–H activation/annulation as resource-economic approach. E = heteroatom. TM = transition metal.

Alkynes are key substrates in molecular synthesis. Due to their versatile reactivity, they enable a wide array of transformations, including cycloadditions, coupling reactions, and hydrofunctionalizations, among others.^1^ Hence, access to compounds is provided that are essential for a broad spectrum of applications, ranging from materials sciences to drug development and crop protection. In this context, the transition-metal catalyzed alkyne annulation is a powerful tool for the assembly of diverse heterocyclic compounds as it allows for the expedient construction of intricate molecular frameworks.^2^ However, especially Larock-type heteroannulations face limitations, including the need for pre-functionalized substrates, expensive catalysts, and harsh reaction conditions. While alkyne annulations *via* C–H activation offer improvements in terms of step economy, significant obstacles remain, as the use of toxic heavy metal salts as oxidant is often involved. Consequently, these drawbacks have led to a strong demand for more sustainable strategies (Fig. 1a). As a consequence, aerobic transition metal-catalyzed C–H activation was introduced for the construction of heterocycles, exhibiting improved atom economy with water as the sole byproduct (Fig. 1b). Thus, in 2015, Ackermann described the aerobic ruthenium-catalyzed C–H annulation for the assembly of isocoumarins.^3^ Despite major advances, such oxidase catalysis had thus far been limited to toxic and expensive precious transition metals. In sharp contrast, in 2016, Ackermann reported on aerobic cobalt-catalyzed C–H alkyne annulations to access versatile isoquinolones.^4^ While representing key progress, aerobic transition metal-catalysis was characterized by major drawbacks, including (a) fixed redox potential with limited tunability,^5^ (b) safety hazards associated with the use of molecular oxygen with flammable solvents.^6^ Thus, for industrial processes the limiting oxygen concentration (LOC), which defines the minimum partial pressure of oxygen that supports a combustible mixture, prohibits the broad implementation.^6^ In contrast, the advent of metallaelectro-catalyzed C–H activation, which combines transition-metal catalyzed C–H activation and electrochemistry, offers an inherently safe and sustainable approach to construct valuable organic molecules (Fig. 1c).^7^ Importantly, this synergistic strategy offers a scalable approach to harness renewable forms of energy for a green hydrogen economy through the cathodic hydrogen evolution reaction (HER).^8^ Whereas, in metallaelectro-catalyzed C–H activation electricity – protons and electrons – is employed as a “traceless-oxidant”, obviating the formation of stoichiometric waste generated from chemical oxidants.^9^ Herein, we thus summarize the rapid recent evolution of metalla-electrocatalysis for alkyne annulations until August 2024.

## 2 4d and 5d metallaelectro-catalyzed alkyne annulations

### 2.1 Rhodaelectro-catalyzed C–H activation

**Scheme 1:**
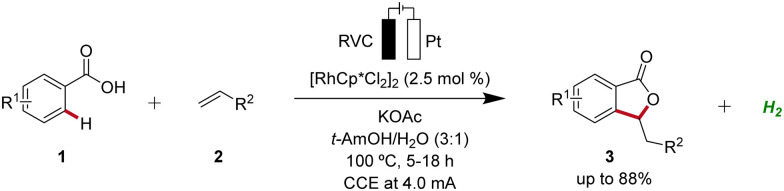
Assembly of isobenzofuranones 3 enabled by the first rhodaelectro-catalyzed C–H activation.

Rhodium catalysis represents a powerful and versatile approach to chemical synthesis, offering high efficiency and selectivity across a wide range of transformations.^10^ Pioneering work in the field of rhodaelectro-catalyzed C–H activation was accomplished by Ackermann in 2018 (Scheme 1).^11^ Here, the electro-oxidative C–H activation of weakly coordinating benzoic acids 1 for the assembly of versatile isobenzofuranones 3 was described.

**Scheme 2:**
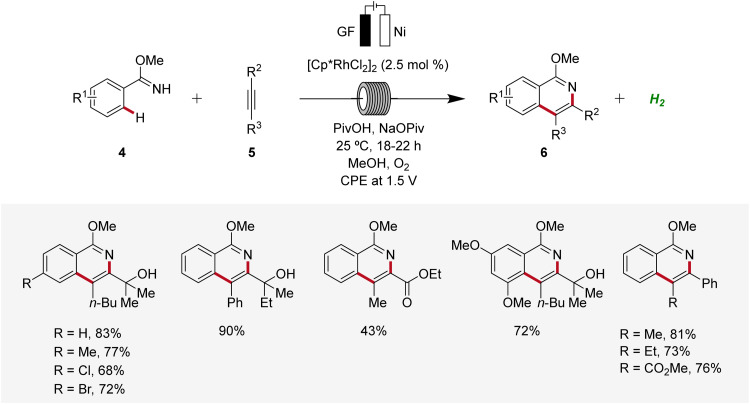
Flow rhodaelectro-catalyzed alkyne annulations for the synthesis of isoquinolines 6.

Subsequently, in 2019, Ackermann established a user-friendly and scalable flow rhodaelectro-catalyzed alkyne annulation for the synthesis of isoquinolines 6 (Scheme 2).^12^ The electrocatalysis proved amenable to differently substituted aryl imidates 4 under flow-electrochemical conditions. This electrocatalytic C–H/N–H alkyne annulation exhibited high levels of functional group tolerance and remarkable regioselectivity with unsymmetrical alkynes 5. Moreover, the electro-flow approach was suitable for intramolecular C–H/N–H functionalization, providing direct access to azo-tetracycles.^12^

**Scheme 3:**
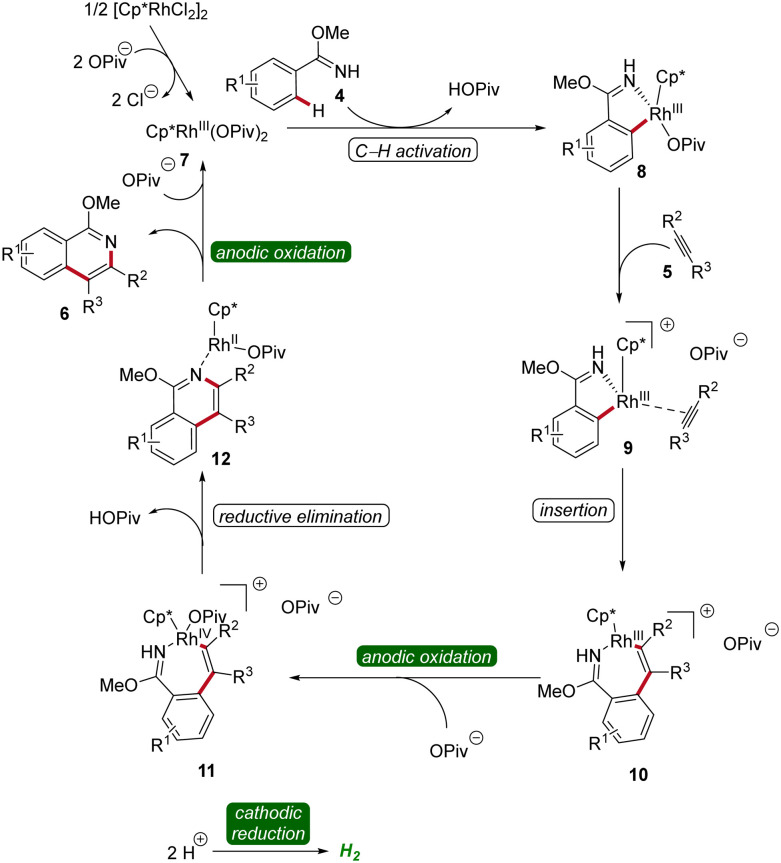
Mechanism of flow rhodaelectro-catalyzed alkyne annulations for the synthesis of isoquinolines 6.

The mechanism of rhoda-electrocatalysis was investigated in detail, employing the isolation and characterization of relevant organometallic intermediates, *in operando* kinetic studies, cyclic voltammetric investigations, and DFT analyses. Thus, the pre-catalyst [Cp*RhCl_2_]_2_ first undergoes ligand exchange with NaOPiv to form the monomeric Cp*Rh(OPiv)_2_7. This complex then is coordinated by the imidate 4, followed by the formation of the rhoda(iii)-cycle 8 through C–H activation. Subsequent coordination of the alkyne 5 and migratory insertion result in the generation of the rhodium(iii) heptacycle 10. Under the electrochemical conditions, the formation of product 6 is promoted by an oxidation-induced reductive elimination involving the anodic oxidation of the rhoda(iii)-cycle 10 to generate rhodium(iv) intermediate 11 (Scheme 3).^12^

**Scheme 4:**
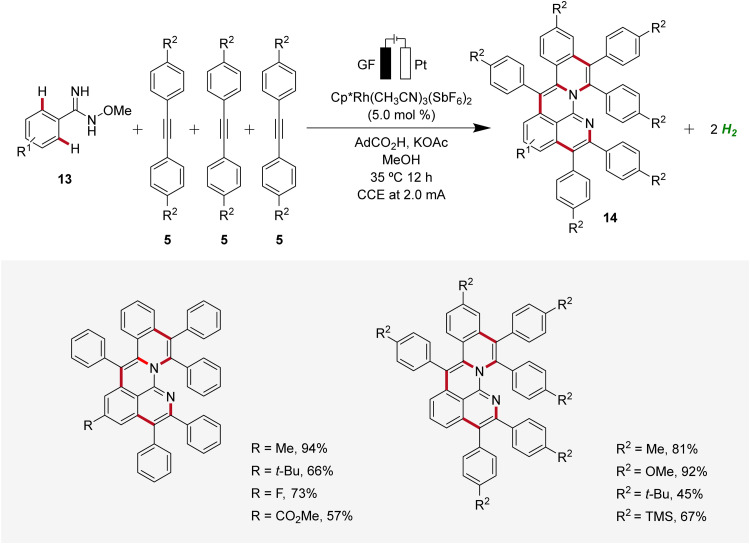
Electrochemical synthesis of aza-polycyclic aromatic hydrocarbons 14*via* rhodaelectro-catalyzed domino C–H annulations.

In 2020, Ackermann reported an unique one-step electrochemical assembly of aza-polycyclic aromatic hydrocarbons 14 (aza-PAH) using rhodaelectro-catalyzed domino C–H annulations (Scheme 4).^13^ The reaction of amidoximes 13 and alkynes 5 resulted in the desired aza-PAHs 14*via* threefold C–H activations with high levels of regioselectivity. The feasibility of this electrocatalysis was proven by scalability, user-friendly setup, and mild reaction conditions. Hence, the electrocatalytic transformation was efficiently established in an undivided cell setup with ample scope and significant levels of functional group tolerance.^13^

**Scheme 5:**
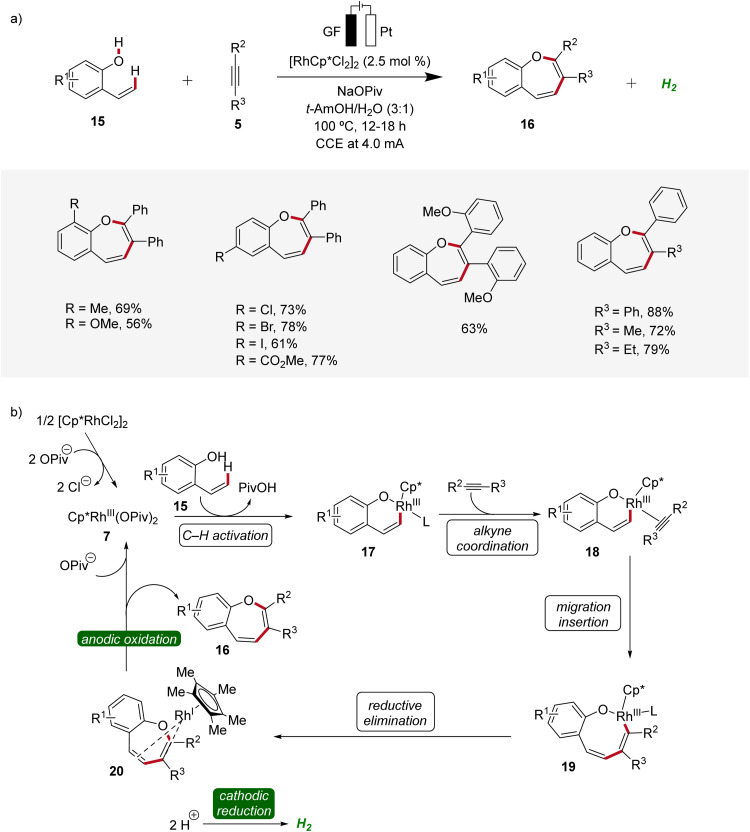
Versatility and mechanism of the rhodaelectro-catalyzed synthesis of benzoxepines 16.

Recently, metallaelectro-catalyzed reactions have provided efficient routes for the construction of various five- and six-membered heterocyclic ring structures *via* formal [3 + 2] or [4 + 2] cycloadditions.^14^ In 2021, Ackermann reported the first rhodaelectro-catalyzed [5 + 2] cycloaddition reactions for the synthesis of benzoxepine motifs 16 using 2-vinylphenols 15 and alkynes 5 (Scheme 5).^15^ This rhodium(iii/i)-catalyzed annulation reaction was amenable to diversely functionalized 2-vinylphenols 15 and alkynes 5, demonstrating a broad substrate scope and functional group tolerance. Detailed mechanistic studies revealed a facile C–H rhodation under a rhodium(iii/i) regime. Furthermore, a benzoxepine-coordinated rhodium(i) sandwich complex 20 could be isolated, which could further be confirmed as a crucial intermediate of the devised electrocatalysis.^15^

**Scheme 6:**
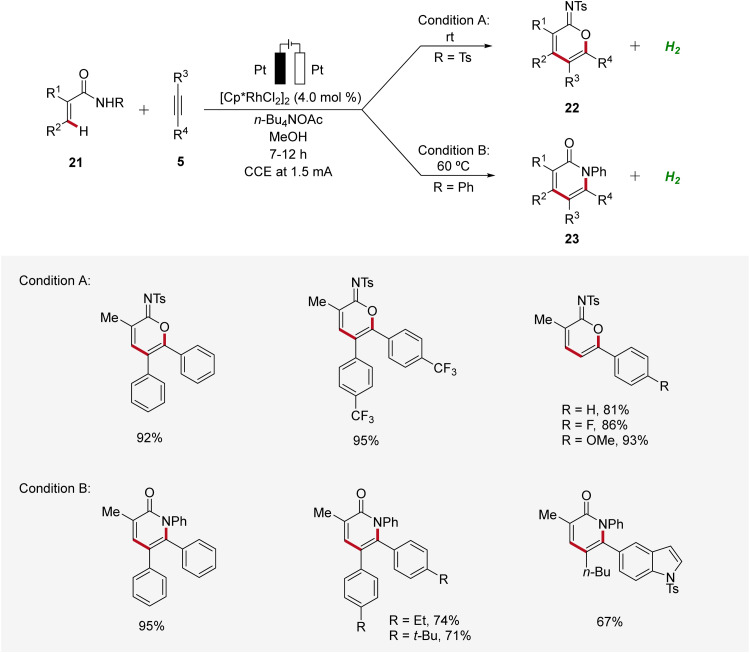
Synthesis of imidates 22 and α-pyridones 23 enabled by divergent rhodaelectro-catalyzed vinylic C–H annulation.

In 2021, Mei established the vinylic C–H annulation of acrylamides 21 with alkynes 5 using divergent rhodaelectro-catalysis (Scheme 6).^16^ Various cyclic imidates 22 and α-pyridones 23 were synthesized by varying the *N*-substituent of acrylamides 21 in an undivided cell using mild reaction conditions. The electrocatalysis proceeds for both reaction pathways with excellent regioselectivity using unsymmetrical internal or terminal alkynes 5.

**Scheme 7:**
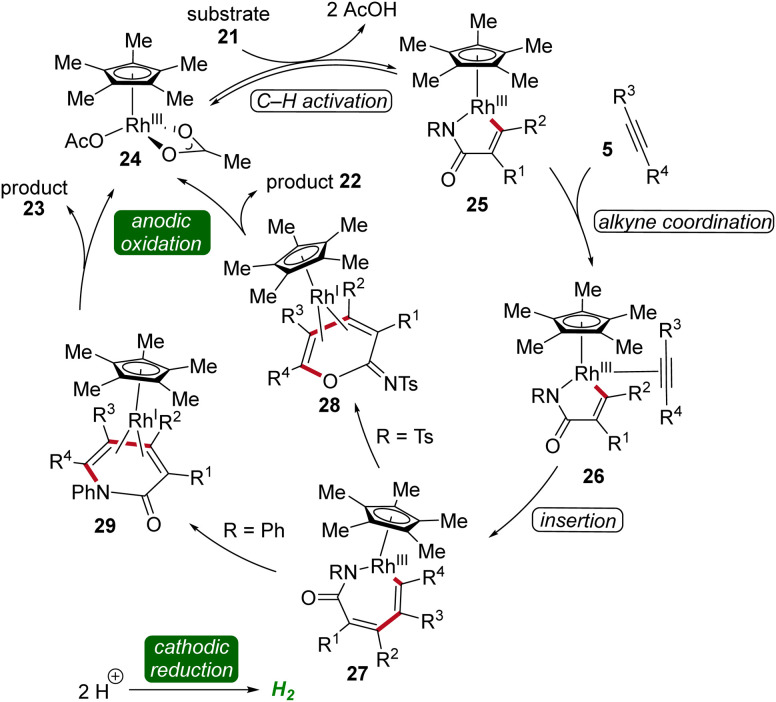
Plausible catalytic cycle for the assembly of imidates 22 and α-pyridones 23.

Cyclic voltammetric analysis and kinetic isotopic effect studies have elucidated the mechanism of this rhodaelectro-catalyzed vinylic C–H annulation. The seven-membered rhoda(iii)-cycle 27 is formed by C–H activation followed by insertion of alkyne 5. This intermediate can undergo two distinct pathways: depending on the electronic nature of the *N*-substituent of the acrylamide 21 either an ionic stepwise pathway that generates intermediate 28, which further yields the cyclic imidates 22, or directly a reductive elimination, generating intermediate 29, which leads to the formation of pyridones 23 takes place (Scheme 7).^16^

**Scheme 8:**
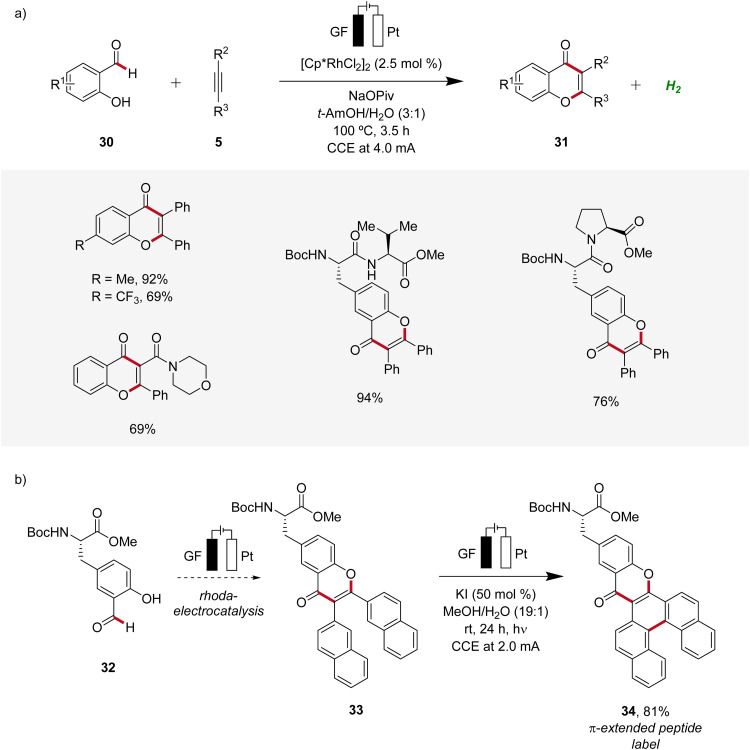
Versatility of rhodaelectro-catalyzed alkyne annulations for the synthesis of chromones 31 and its application to introduce fluorescent labels 34.

In 2021, Ackermann developed a rhodaelectro-catalyzed formyl C–H activation (Scheme 8).^17^ This strategy enabled the direct synthesis of various chromones 31 from hydroxybenzaldehydes 30. Notably, despite benzaldehydes generally being considered oxidation-sensitive, the identified mild reaction conditions for the rhoda-electrocatalysis allowed for an applicability with a wide range of substrates including peptides (Scheme 8a). Moreover, it was demonstrated that from the obtained chromone 33 π-extended peptide labels 34 can be accessed through a photoelectrochemical process (Scheme 8b).^17^

**Scheme 9:**
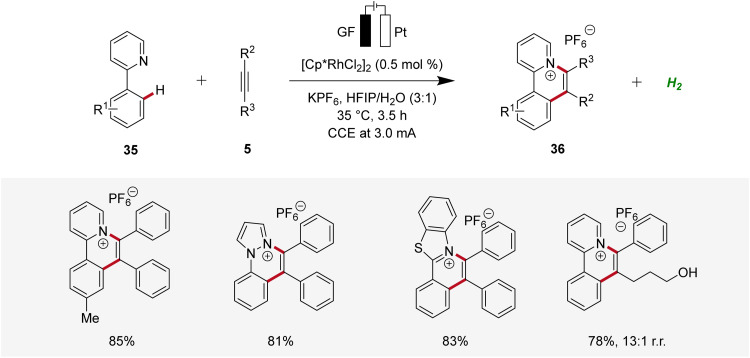
Rhodaelectro-catalyzed C–H annulation to construct cationic polycyclic heteroarenes 36.

In 2021, Zhang described a rhodaelectro-catalyzed C–H annulation for the construction of cationic polycyclic heteroarenes 36 (Scheme 9).^18^ Here, mechanistic studies, including the isolation of organometallic intermediates and cyclic voltammetric analyses, were conducted. Additionally, the regioselectivity in the annulation process was elucidated through detailed computational studies.^18^

**Scheme 10:**
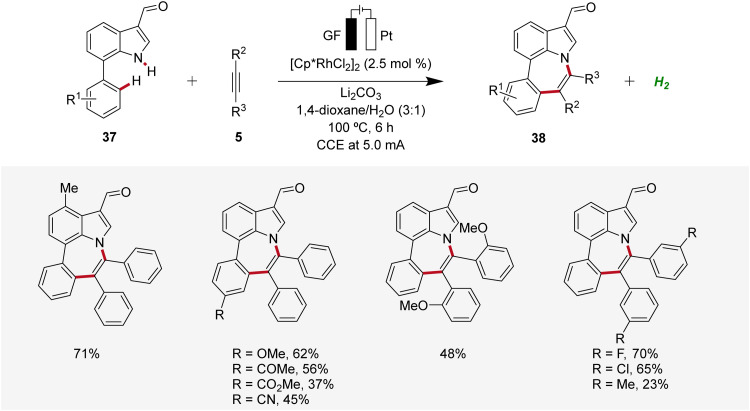
Rhodaelectro-catalyzed [5 + 2] C–H/N–H annulation reaction for the construction of azepino[3,2,1-*hi*]indoles 38.

In 2022, Ackermann, Huang, and Ni reported a rhodaelectro-catalyzed [5 + 2] C–H/N–H annulation using 7-phenylindoles 37 with alkynes 5 in an undivided cell to construct azepino[3,2,1-*hi*]indoles 38 (Scheme 10).^19^ This electrocatalysis exhibited a broad substrate scope with ample functional group tolerance and gram scalability through flow electrocatalysis. Thus, 7-phenylindoles 37 substituted at different positions as well as *ortho*-, *meta*-, or *para*-substituted diphenylacetylenes 5 proved to be compatible.^19^

**Scheme 11:**
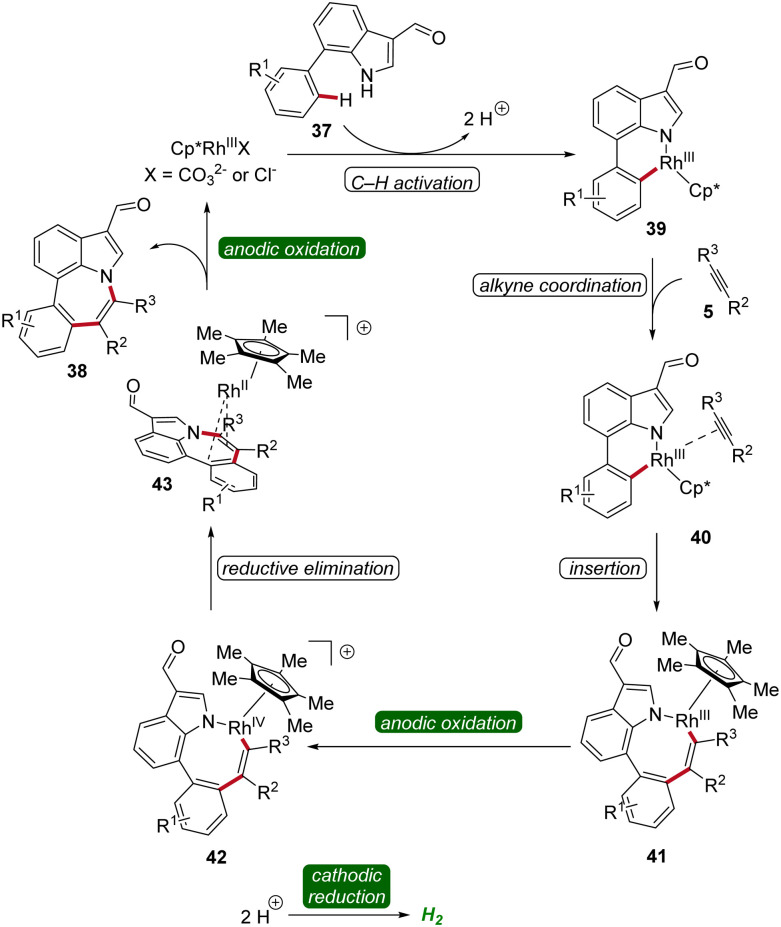
Mechanism of rhodaelectro-catalyzed [5 + 2] C–H/N–H annulation reaction for the assembly of azepino[3,2,1-*hi*]indoles 38.

A reaction mechanism was proposed derived from deuterium-labeling studies, cyclic voltammetric analyses, and X-ray photoelectron spectroscopy studies. Based on these findings, the formation of the six-membered rhoda(iii)-cycle 39 through C–H activation was postulated. A migratory insertion with coordinated alkyne 5 then occurs, leading to the eight-membered rhoda(iii)-cycle 41. Finally, an oxidation-induced reductive elimination *via* a rhodium(iii/iv/ii) pathway facilitates the release of the azepino[3,2,1-*hi*]indole product 38 (Scheme 11).^19^

**Scheme 12:**
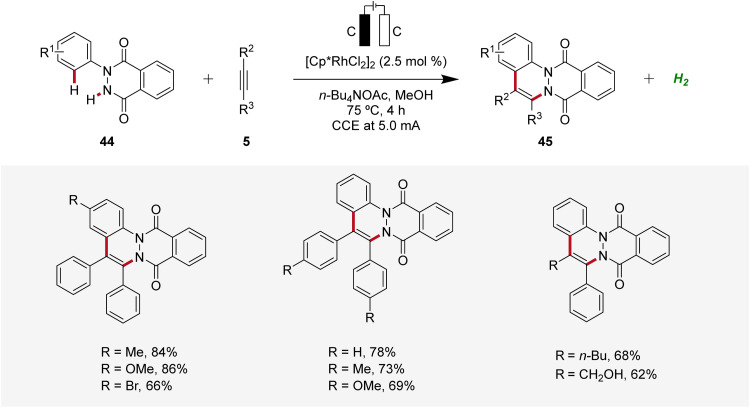
Rhodaelectro-catalyzed C–H/N–H annulation for the synthesis of cinnolines 45.

Similarly, in 2022, a rhodaelectro-catalyzed [4 + 2] C–H annulation was reported by Roy for the synthesis of cinnolines 45 (Scheme 12).^20^ The C–H/N–H annulation of arylhydrophthalazinediones 44 with alkynes 5 using precatalyst [Cp*RhCl_2_]_2_ in an undivided cell under galvanostatic conditions afforded efficiently the desired cinnolines 45. The robustness and versatility of the developed method was tested by employing diversely decorated 2-aryl-3-hydrophthalazinediones 44 as well as symmetrical and unsymmetrical internal alkynes 5, while the desired products 45 were furnished in good to excellent yields. However, terminal alkynes were not compatible. Cyclic voltammetry and differential pulse voltammetry experiments revealed the formation of the annulated products 45 through a Rh(iii/i) and Rh(iii/iv) pathway.^20^

**Scheme 13:**
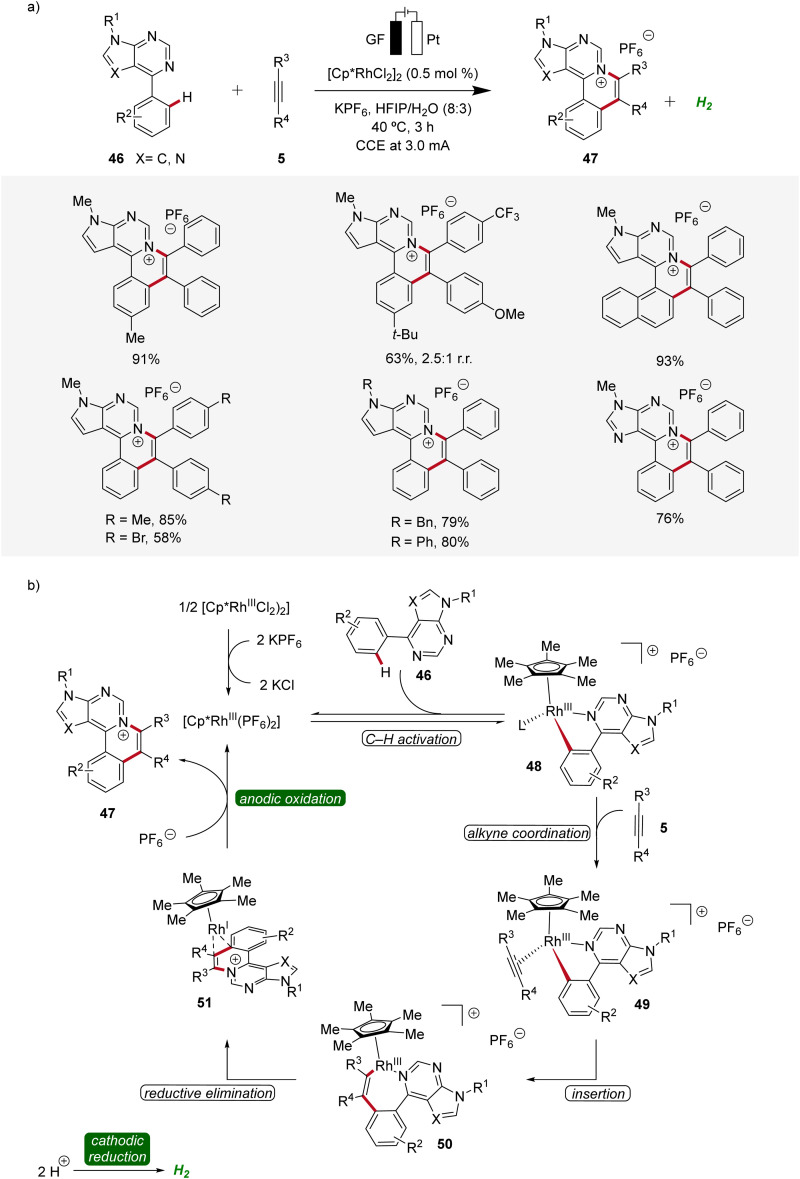
Versatility and mechanism of rhodaelectro-catalyzed [4 + 2] C–H annulation.

Likewise, a rhodaelectro-catalyzed [4 + 2] C–H activation/annulation with internal alkynes 5 was reported by Ling in 2022 (Scheme 13).^21^ This expedient strategy provided a new series of polycyclic (7-deaza)purinium salts 47 in excellent yields and proved to be compatible with various substitution patterns on both the (7-deaza)purine 46 as well as the alkyne 5. Mechanistic studies employing cyclic voltammetry demonstrated that the coordination of 46 to the Cp*Rh(iii) catalyst and successive cyclometallation gives rhoda(iii)-cycle 48, which upon migratory insertion with alkyne 5 and subsequent reductive elimination delivers the rhodium(i) sandwich complex 51. By anodic oxidation of complex 51 the annulated product 47 is released and the catalytically competent rhodium(iii) is regenerated (Scheme 13).^21^

**Scheme 14:**
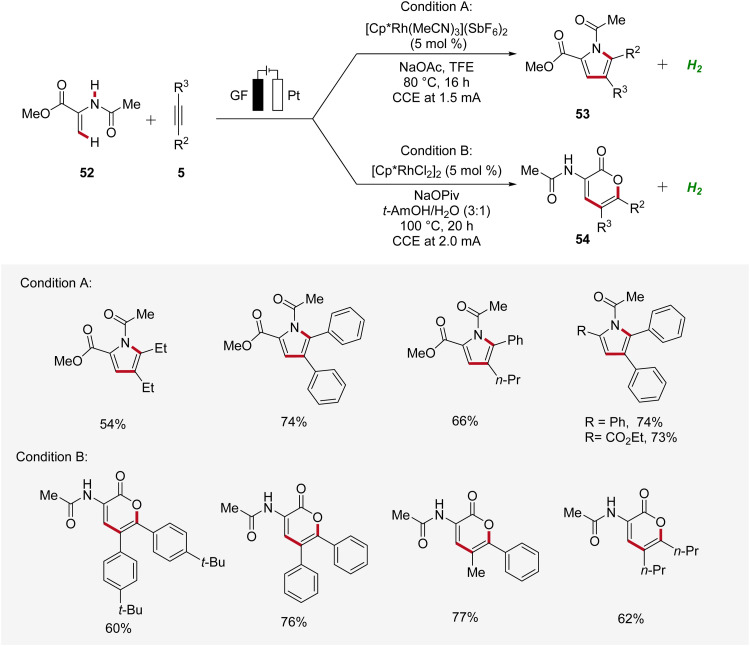
Bifurcated rhodaelectro-catalyzed C–H annulation strategy for the synthesis of pyrroles 53 and lactones 54.

Recently, Ackermann accomplished rhodaelectro-catalyzed C–H annulations using enamides 52 and alkynes 5 in an user-friendly undivided cell setup (Scheme 14).^22^ Interestingly, a bifurcated reaction pathway was uncovered, where the solvent system was identified as crucial factor in controlling the chemo-selectivity. Thus, through the rational choice of the reaction medium, the product formation between pyrroles 53 and lactones 54 could be switched. This example demonstrates how the ability to control chemo-selectivity broadens synthesis possibilities and allows access to a wider range of heterocyclic structures.^22^

**Scheme 15:**
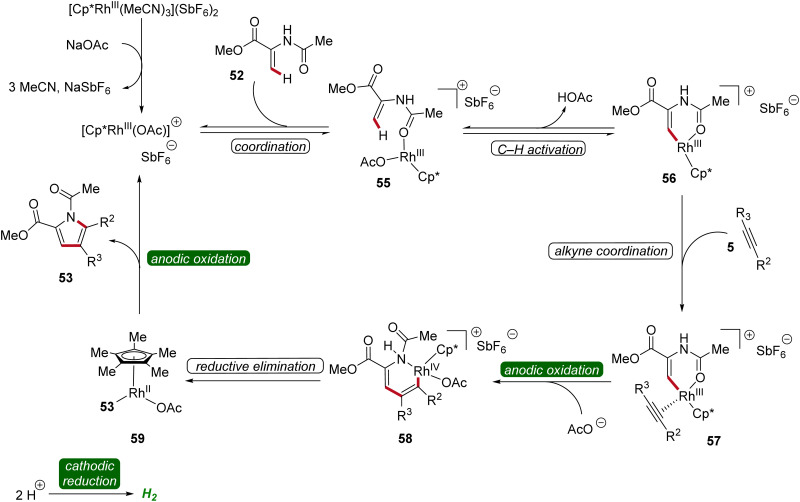
Plausible catalytic cycle for the bifurcated rhodaelectro-catalyzed C–H annulation leading to pyrroles 53.

The bifurcated rhodaelectro-catalysis to construct pyrroles 53 or lactones 54 involves a multi-step reaction mechanism (Scheme 15). Initially, the catalytically active Cp*Rh(iii) species is formed followed by the coordination of enamide 52, yielding intermediate 55. Next, C–H activation takes place to form rhoda(iii)-cycle 56. Thereafter, migratory insertion of alkyne 5 and anodic oxidation results in the formation of rhodium(iv) species 58, promoting a reductive elimination to form intermediate 59. The active rhodium(iii) catalyst is then regenerated through anodic oxidation, ultimately releasing product 53. Regarding the chemo-divergence, it is proposed that the cathodic hydrogen evolution reaction (HER) promotes the ester hydrolysis when an aqueous medium is employed, initiating the divergent catalytic scenario primarily involving neutral rhodium intermediates leading to lactones 54.^22^

### 2.2 Iridaelectro-catalyzed C–H activation

**Scheme 16:**
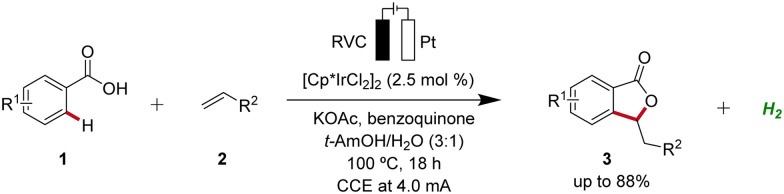
First iridaelectro-catalyzed C–H activation enabled by cooperative action of benzoquinone as redox catalyst.

Iridium-catalyzed C–H activation has emerged as a powerful and versatile methodology in modern organic synthesis.^23^ In 2018, Ackermann developed the first iridaelectro-catalyzed C–H activation, which provided access to various isobenzofuranones 3 from benzoic acids 1 (Scheme 16).^24^ With benzoquinone as redox catalyst an indirect, cooperative electrocatalysis was uncovered.

**Scheme 17:**
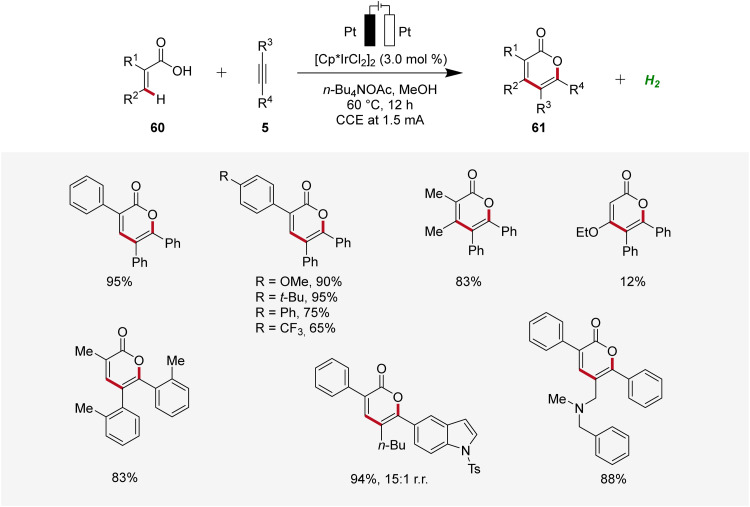
Electrochemical iridium-catalyzed vinylic C–H annulation of acrylic acids 60.

Thereafter, in 2019, Mei developed an iridaelectro-catalyzed C–H annulation of acrylic acids 60 to obtain biorelevant α-pyrones 61 (Scheme 17).^25^ The reaction conditions comprised galvanostatic electrolysis in the presence of a [Cp*IrCl_2_]_2_ pre-catalyst. Various α-substituted acrylic acids 60 and internal alkynes 5 were tolerated, resulting in good to excellent yields of the desired α-pyrones 61. The electrocatalysis demonstrated moderate to excellent regioselectivity with unsymmetrical alkynes 5.^25^

**Scheme 18:**
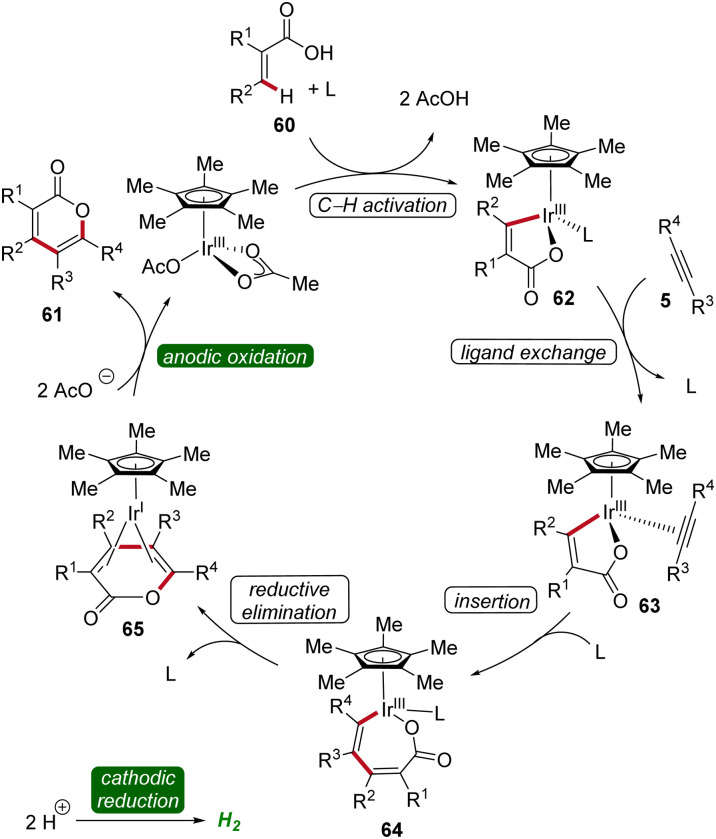
Mechanism of iridium-catalyzed electrochemical vinylic C–H annulation of acrylic acids 60.

The irida-electrocatalysis proceeds in an iridium(iii/i) regime (Scheme 18). The irida(iii)-cycle 62 is formed through carboxylate-assisted C–H activation, followed by coordination and insertion of the alkyne 5. Subsequently, by reductive elimination the sandwich complex 65 is formed, which, through anodic oxidation, releases the product 61.^25^

**Scheme 19:**
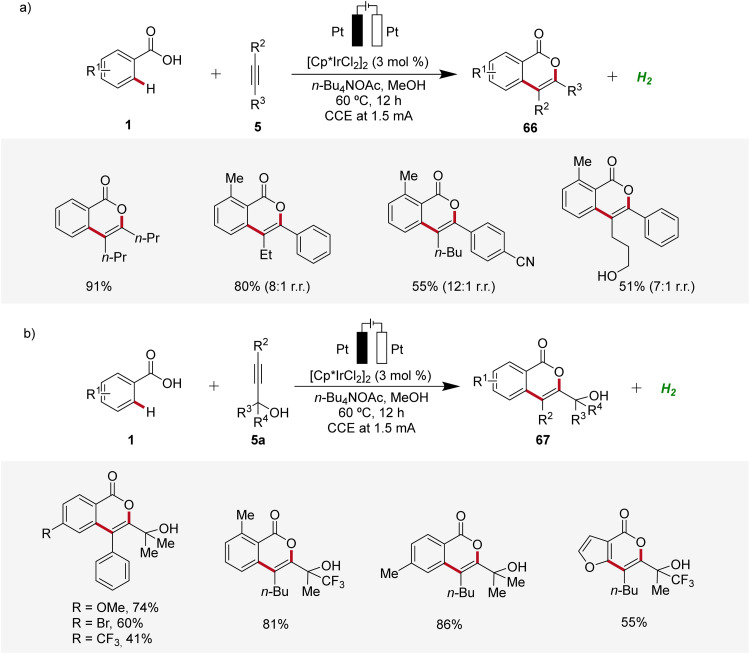
Synthesis of isocoumarin derivatives 66 and 67 through irida-electrocatalysis.

Isocoumarins are known for their significant biological effects and commonly found in natural substances and medicinal compounds.^26^ In 2021, Guo and Mei developed an iridaelectro-catalyzed regioselective annulation of easily accessible aromatic carboxylic acids 1 with internal alkynes 5 to access isocoumarins 66 with moderate to excellent regioselectivity (Scheme 19).^27^ The electrocatalysis demonstrated broad compatibility with various substrates 1 and 5, including dialkyl acetylenes. Mono-substituted benzoic acids 1 with electron-donating and electron-neutral substituents readily reacted in satisfactory yields, while strong electron-withdrawing groups afforded lower yields. However, with more sterically hindered arylalkynes the efficiency is decreased (Scheme 19a). Interestingly, the reaction with *tert*-propargyl alcohols 5a efficiently furnished isocoumarins 67 under identical reaction conditions as a single regioisomer (Scheme 19b).^27^

**Scheme 20:**
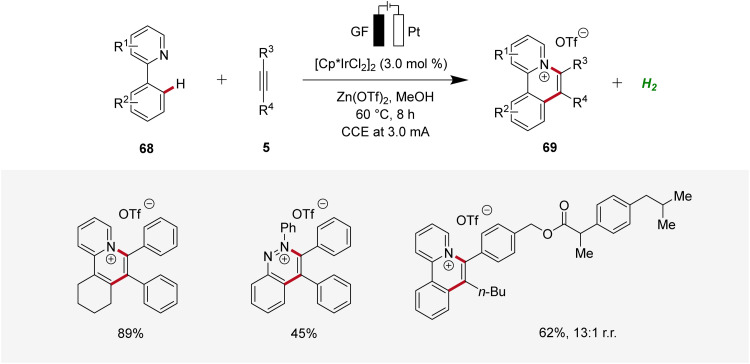
Synthesis of cationic π-extended heteroarenes 69 through irida-electrocatalysis.

Recently, Guo and Yang described an iridaelectro-catalyzed C–H annulation, yielding cationic π-extended heteroarenes 69 (Scheme 20).^28^ The strategy demonstrated a broad substrate scope and was compatible with various *N*-heteroarenes as directing groups, including pyridine and purine derivatives. Additionally, mechanistic studies indicated an iridium(iii/i) regime.^28^

### 2.3 Ruthenaelectro-catalyzed C–H activation

**Scheme 21:**
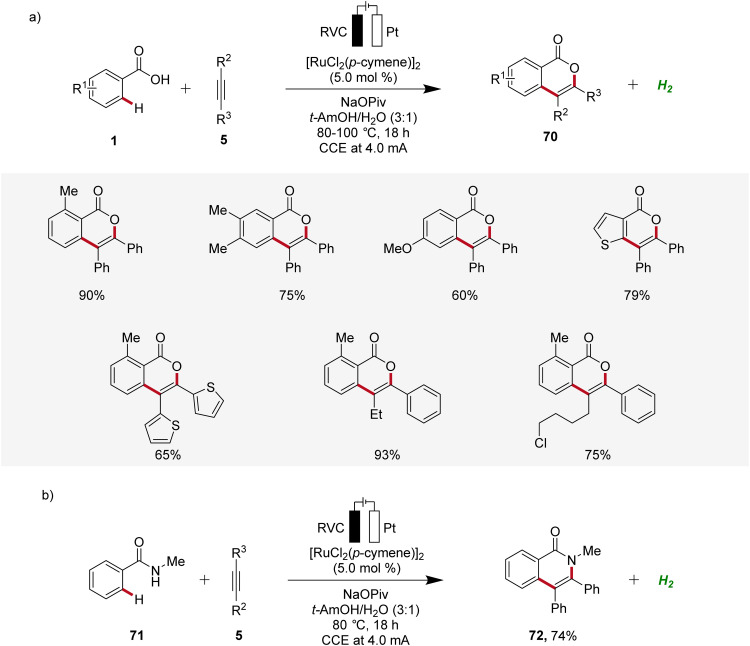
Electro-oxidative ruthenium-catalyzed alkyne annulation to construct (a) isocoumarins 70 and (b) isoquinolones 72.

Ruthenium catalysis is highly attractive due to the exceptional catalytic reactivity of ruthenium, combined with its good availability compared to more expensive transition metals like palladium and rhodium.^29^ In 2018, Ackermann reported the first example of ruthenaelectro-catalyzed C–H activation by weak *O*-coordination for the construction of isocoumarins 70 (Scheme 21).^30^ The reaction involves an *in situ* formed ruthenium(ii) carboxylate catalyst mediating the C–H bond activation in a reaction medium of *tert*-amyl alcohol and water. This ruthena-electrocatalysis proved to be versatile and was amenable to both electron-rich as well as electron-deficient arenes 1 and alkynes 5. Notably, unsymmetrical alkynes 5 reacted to the desired product 70 with high levels of regioselectivity (Scheme 21a). Additionally, the electrocatalysis was also found to be compatible with benzamides 71, to form the corresponding isoquinolones 72 (Scheme 21b).^30^

**Scheme 22:**
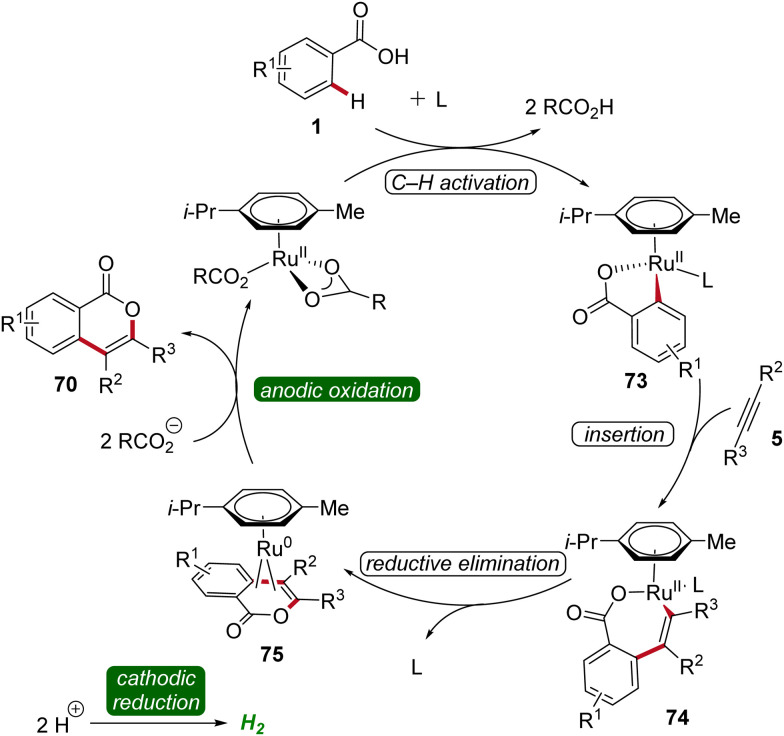
Catalytic cycle for the electro-oxidative ruthenium-catalyzed alkyne annulation by weakly coordinating benzoic acids 1.

Based on detailed mechanistic studies, a plausible catalytic cycle was proposed (Scheme 22). Initially, the *ortho* C–H activation occurs, leading to the formation of the ruthena(ii)-cycle 73. Subsequently, the insertion of alkyne 5 takes place, forming the seven-membered ruthena(ii)-cycle 74, which undergoes reductive elimination to produce the ruthenium(0) sandwich complex 75. This complex is then anodically oxidized, releasing product 70 and regenerating the catalytically competent ruthenium(ii) carboxylate species, while cathodic reduction generates molecular hydrogen being the sole stoichiometric byproduct (Scheme 22).^30^

**Scheme 23:**
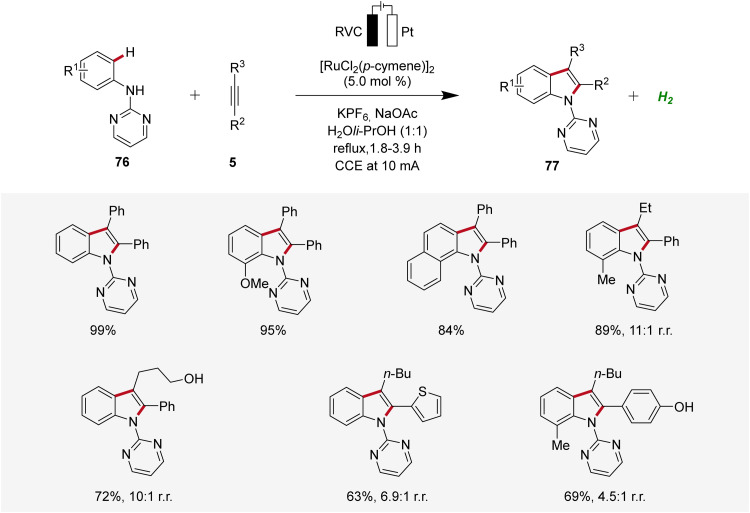
Ruthenaelectro-catalyzed C–H/N–H annulation for the synthesis of indoles 77.

Concurrently, Xu developed a ruthenaelectro-catalyzed C–H annulation of anilines 76 with alkynes 5 in an undivided cell under galvanostatic electrolysis (Scheme 23).^31^ The electrocatalysis allowed access to indoles 77 with diverse functional groups in good to excellent yields. However, substrates with highly sterically hindered functional groups exhibited diminished regioselectivity and reactivity.^31^

**Scheme 24:**
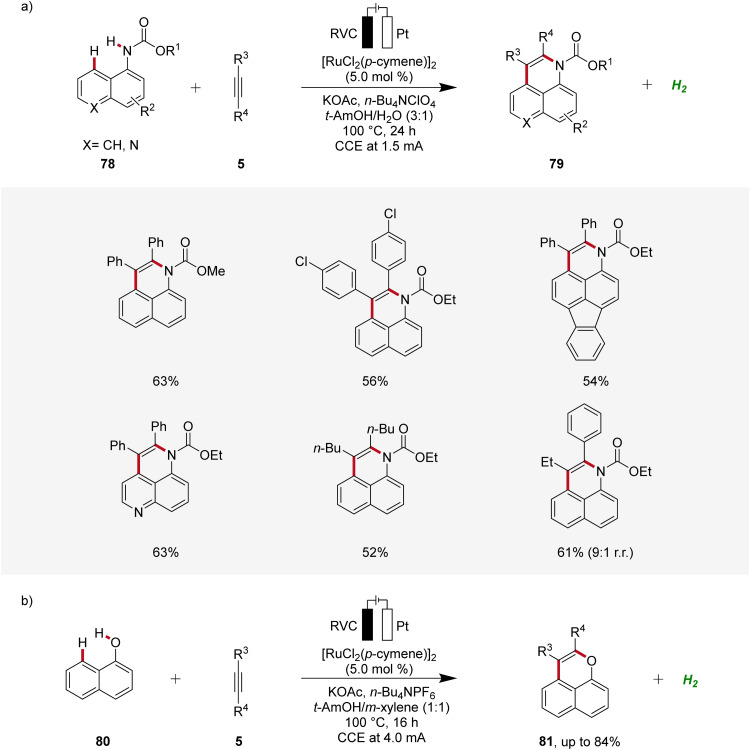
Ruthenaelectro-catalyzed *peri*-selective C–H alkyne annulations to access benzoquinolines 79 and pyrans 81.

In 2018, Ackermann established an electrochemical *peri*-selective C–H alkyne annulation of aryl carbamates 78 and naphthols 80 using ruthenium-catalysis (Scheme 24).^32^ Here, electrochemical conditions for facilitating both C–H/N–H and C–H/O–H annulations were identified. The versatility of this approach was assessed by varying the functional groups on both substrates demonstrating excellent site-, regio-, and chemo-selectivity. The strategy provided access to diverse benzoquinoline derivatives 79 and pyrans 81 in a step-economical manner with high efficacy and selectivity.^32^

**Scheme 25:**
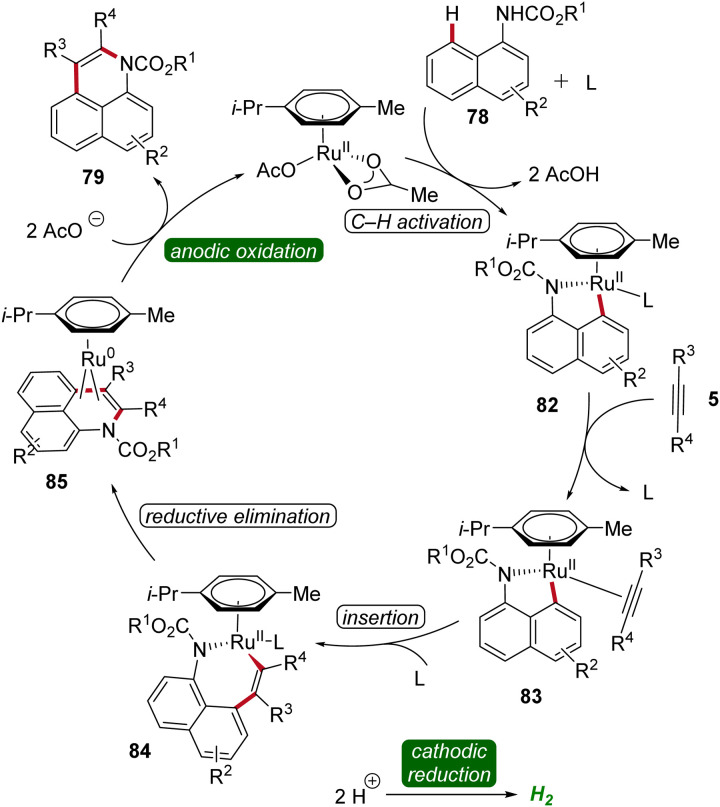
Catalytic cycle for the ruthenaelectro-catalyzed alkyne annulation of arylcarbamates 78.

Based on detailed mechanistic studies, a possible catalytic cycle was proposed (Scheme 25). The catalytic cycle begins with organometallic C–H activation, generating a ruthena(ii)-cycle 82. Migratory alkyne insertion then forms a seven-membered ruthena(ii)-cycle 84, which undergoes reductive elimination to produce a ruthenium(0)-sandwich complex 85. The anodic oxidation of complex 88 results in the desired product 79.^32^

**Scheme 26:**
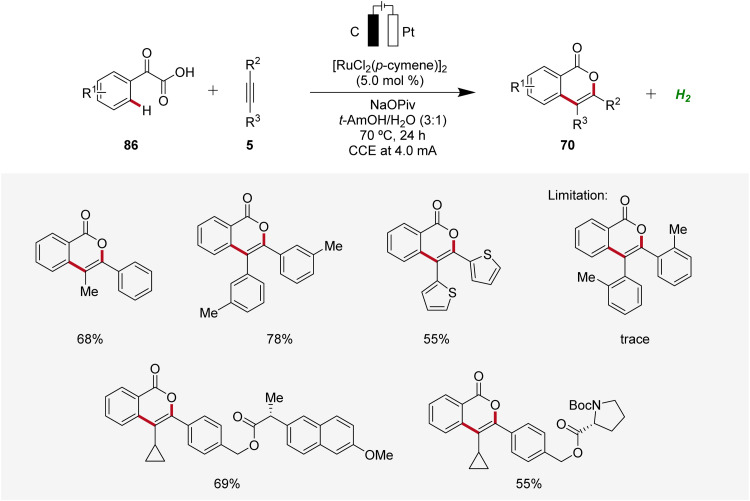
Decarboxylative ruthenaelectro-catalyzed C–H annulation to access isocoumarins 70.

In 2019, Li and He likewise employed ruthenaelectro-catalysis to access isocoumarins 70 (Scheme 26).^33^ Here, an electrochemical decarboxylative C–H annulation strategy involving arylglyoxylic acids 86 and internal alkynes 5 was devised for the construction of isocoumarins 70. This regime was applicable with both symmetrical and unsymmetrical internal alkynes 5 showing high levels of regioselectivity. However, sterically congested alkynes 5 as well as electron-withdrawing functional groups on the arylglyoxylic acid 86 resulted in low efficiency of the electrocatalysis.^33^

**Scheme 27:**
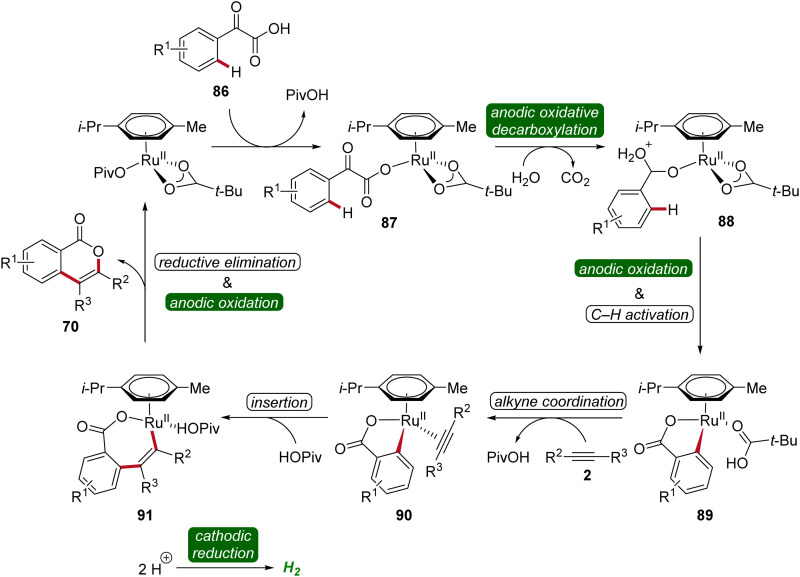
Mechanism of the decarboxylative ruthenaelectro-catalyzed C–H annulation.

To gain mechanistic insights of the decarboxylative ruthena-electrocatalysis, ^18^O-labeled isotope and kinetic isotope effect experiments were conducted. Here, a cooperative action of the anodic decarboxylation and C–H activation was found. The reaction initiates with the carboxyl group of the arylglyoxylic acid 86 coordinating to the active ruthenium(ii) carboxylate species, leading to the formation of intermediate 87. Subsequently, this intermediate undergoes anodic single-electron oxidation to promote a decarboxylation and hydration to yield intermediate 88. Next, further anodic oxidation along with C–H activation leads to ruthena(ii)-cycle 89 and the migratory insertion of alkyne 5 to generate the seven-membered ruthena(ii)-cycle 91 is realized. Lastly, reductive elimination takes place, resulting in the formation of desired product 70 and the active ruthenium(ii) carboxylate species is regenerated by anodic oxidation (Scheme 27).^33^

**Scheme 28:**
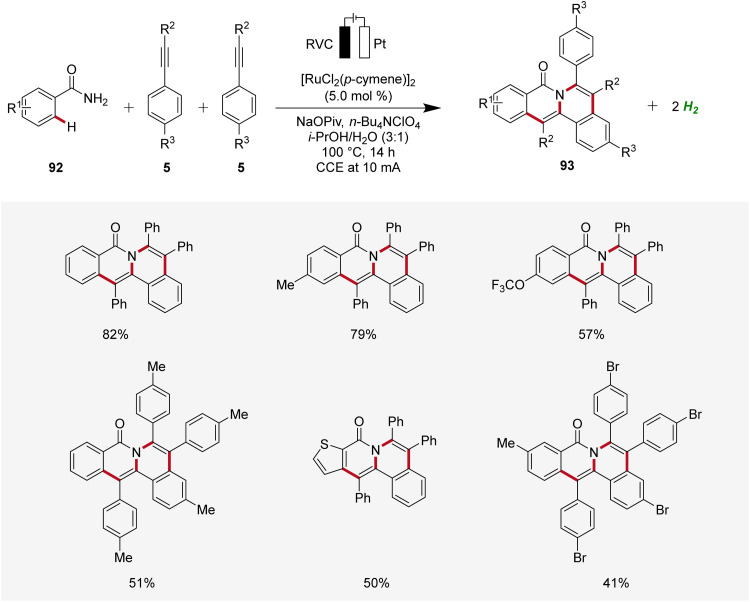
Ruthenaelectro-catalyzed C–H annulation for the chemoselective synthesis of polycyclic isoquinolinones 93.

In 2019, Tang developed an electrocatalytic method for synthesizing polycyclic isoquinolinones 93 through double C–H activation (Scheme 28).^34^ The reaction was effective using a simple undivided cell under galvanostatic electrolysis and was compatible with a wide range of benzamides 92 and alkynes 5 yielding the desired fused products 93 with medium to excellent yields. The high site-selectivity of the electrocatalysis was further demonstrated by using *meta*-substituted benzamides 92.^34^

**Scheme 29:**
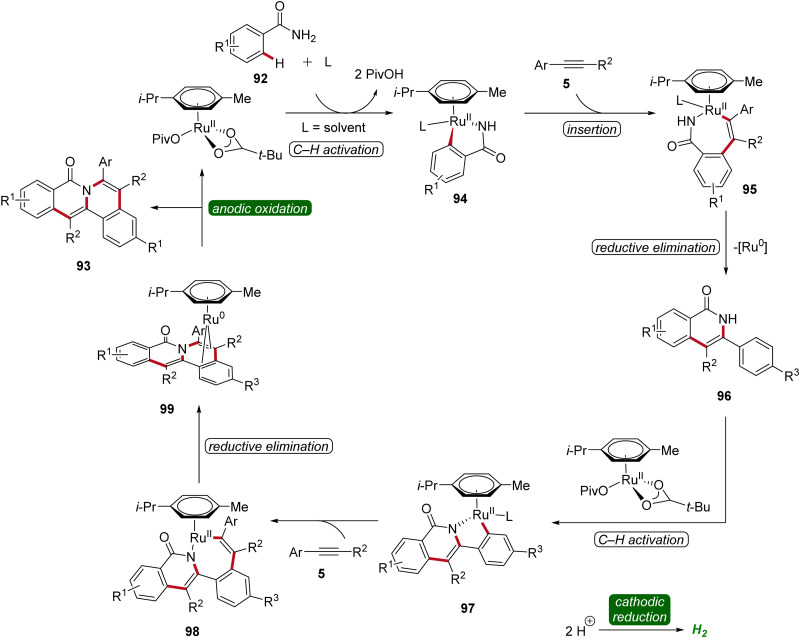
Simplified catalytic cycle for the ruthenaelectro-catalyzed double C–H activation.

The initial step of the proposed mechanism involves the formation of ruthena(ii)-cycle 94 through C–H activation. Subsequently, the insertion of alkyne 5 leads to intermediate 95, which then undergoes reductive elimination to furnish 96. This is followed by a second C–H activation event, resulting in the formation of yet another cyclometallated intermediate 97. Through a sequence involving the insertion of a second alkyne 5 and subsequent reductive elimination, the ruthenium(0) sandwich complex 99 is formed. Ultimately, product 93 is released from complex 99 through anodic oxidation (Scheme 29).^34^

**Scheme 30:**
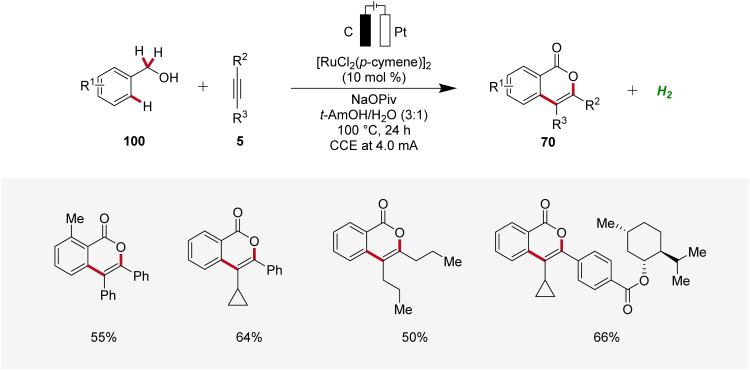
Ruthenaelectro-catalyzed C–H annulations for the synthesis of isocoumarins 70 from benzylic alcohols 100.

Li applied the ruthenaelectro-catalyzed C–H annulation strategy for the synthesis of isocoumarin cores 70 from primary benzylic alcohols 100 (Scheme 30).^35^ Notably, this regime allowed benzylic alcohols 100 to act as weakly directing group precursors to acquire isocoumarins 70*via* multiple C–H functionalizations. The electrocatalysis displayed high regio- and site-selectivity with a broad substrate scope. In contrast to internal alkynes 5, terminal alkynes were not found to be compatible with this strategy.^35^

**Scheme 31:**
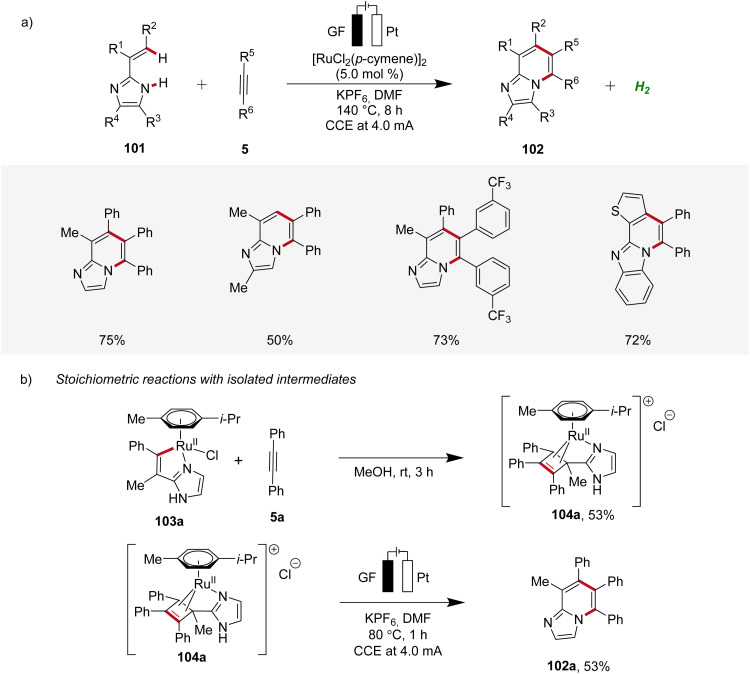
Ruthenaelectro-catalyzed synthesis of bridgehead *N*-fused [5,6]-bicyclic heteroarenes 102.

In 2020, Ackermann further demonstrated a ruthena-electrocatalysis for the assembly of diverse bridgehead *N*-fused [5,6]-bicyclic heteroarenes 102 from imidazoles 101 with alkynes 5, involving an oxidation-induced reductive elimination pathway (Scheme 31).^36^ The versatility of this strategy was explored with various imidazole 101 and alkyne 5 substrates decorated with a range of substituents at different positions, amenable to efficiently form the desired products 102. Besides alkenyl imidazoles, also 2-arylimidazoles 101 were applicable (Scheme 31a). Notably, organometallic intermediates 103a and 104a were isolated and employed in stoichiometric reactions, providing strong support for an oxidation-induced reductive elimination within a ruthenium(ii/iii/i) manifold (Scheme 31b).^36^

**Scheme 32:**
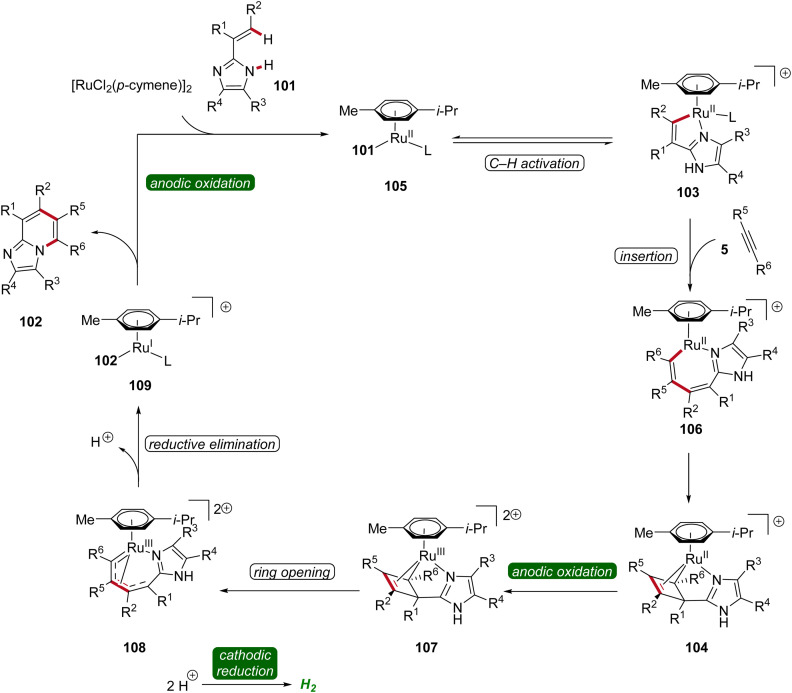
Plausible catalytic cycle for the ruthenaelectro-catalyzed annulation involving azaruthena(ii)-bicyclo[3.2.0]heptadiene intermediates.

Hence, the azaruthena(ii)-bicyclo[3.2.0]heptadiene intermediate 104 formed through alkyne coordination and migratory insertion to the ruthena(ii)-cycle 103 undergoes anodic oxidation to form the ruthenium(iii) complex 107, followed by a pericyclic ring opening to yield 108. Reductive elimination then yields the ruthenium(i) complex 109, which releases the final *N*-fused [5,6]-bicyclic heteroarene 102 (Scheme 32).^36^

**Scheme 33:**
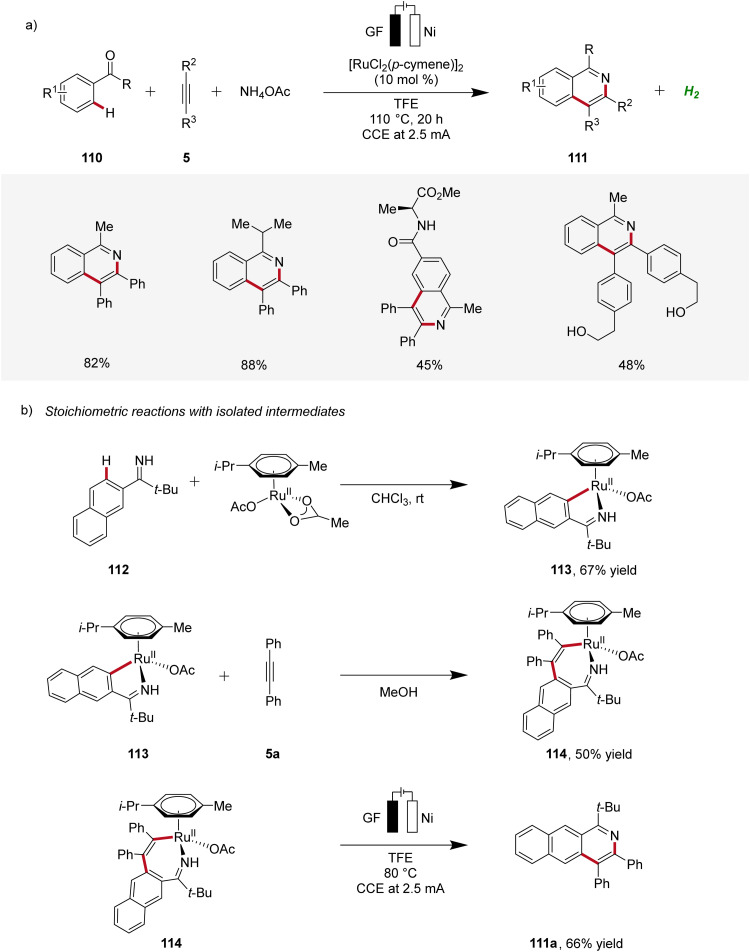
Domino three-component alkyne C–H annulation enabled by ruthena-electrocatalysis.

In 2020, Ackermann reported on a ruthenaelectro-catalyzed domino three-component alkyne C–H annulation, which enabled the expedient construction of isoquinolines 111 from phenones 110, alkynes 5, and ammonium acetate (Scheme 33).^37^ The reaction demonstrated a broad substrate scope, including the compatibility with unprotected alcohol groups. Additionally, relevant cyclometallated ruthenium species 113 and 114 were isolated and their significance for the electrocatalysis was evaluated, supporting a ruthenium(ii/iii/i) pathway.^37^

### 2.4 Osmaelectro-catalyzed C–H activation

**Scheme 34:**
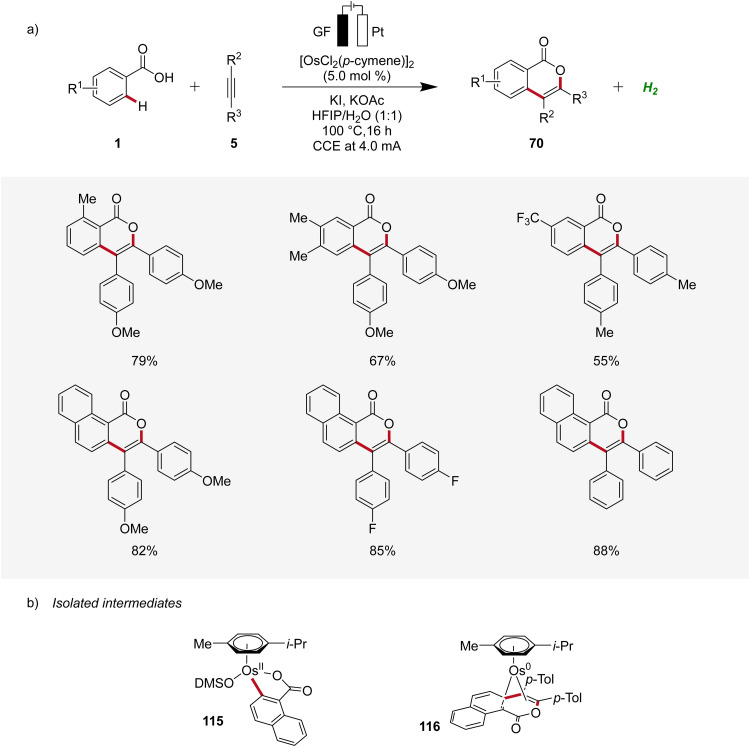
Osmaelectro-catalyzed alkyne annulation by weakly coordinating acids 1.

Osmium, a transition-metal known for its robust reactivity and versatile coordination chemistry, serves as a remarkable catalyst in various redox processes.^38^ In 2021, Ackermann described the first osmaelectro-catalyzed C–H activation (Scheme 34a).^39^ The strategy allowed expedient access to isocoumarins 70 from benzoic acids 1 and alkynes 5 with a broad tolerance to functional groups. Furthermore, systematic reaction monitoring by NMR spectroscopy and HR-ESI-mass spectrometry provided support for an osmium(ii/0) manifold, while key organometallic intermediates 115 and 116 were isolated and studied (Scheme 34b).^39^

### 2.5 Enantioselective 4d metallaelectro-catalyzed alkyne annulations

**Scheme 35:**
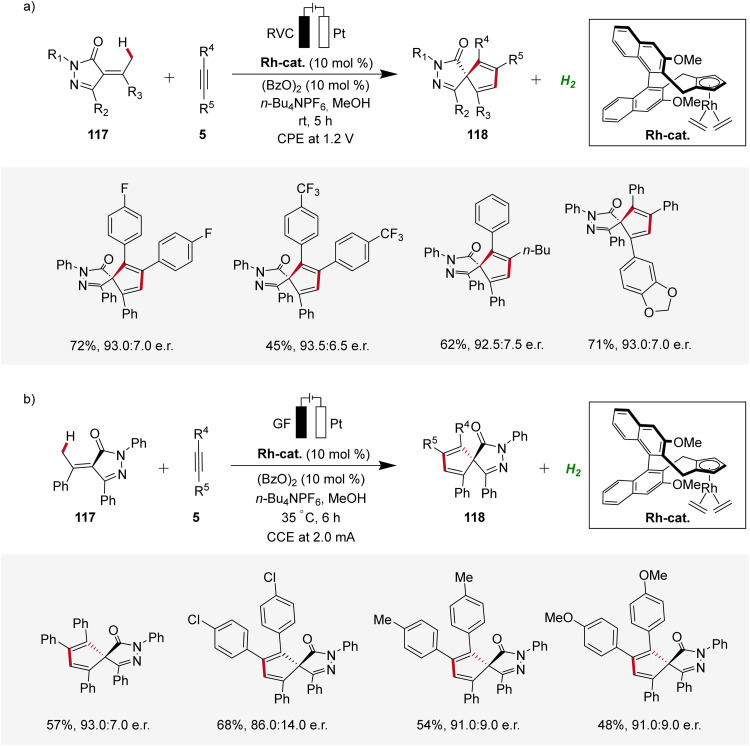
Enantioselective rhodaelectro-catalyzed C–H annulations for the synthesis of spiropyrazolones 118.

In recent years, enantioselective electrocatalysis has emerged as an increasingly versatile tool for the assembly of complex molecules.^40^ Pioneering work in the domain of enantioselective 4d-metallaelectro-catalyzed C–H activation was contributed by Ackermann in 2020, where palladaelectro-catalyzed C–H alkenylations were disclosed to construct axially chiral biaryls.^41^ Thereafter, in 2021, Mei reported on an enantioselective rhodaelectro-catalyzed C–H annulation for the synthesis of biorelevant spiropyrazolones 118 by reacting α-arylidene pyrazolones 117 with alkynes 5 in an undivided cell under potentiostatic electrolysis (Scheme 35a).^42^ This robust annulation strategy provided access to a variety of chiral spirocycles 118 in decent yields and enantioselectivities.^42^ Concurrently, Ackermann established an enantioselective rhodaelectro-catalyzed strategy for the assembly of chiral spiropyrazolones 118, operating under galvanostatic electrolysis (Scheme 35b). In this study, Ackermann also demonstrated a palladaelectro-catalyzed spiroannulation with alkynes, although without enantioselectivity.^43^

**Scheme 36:**
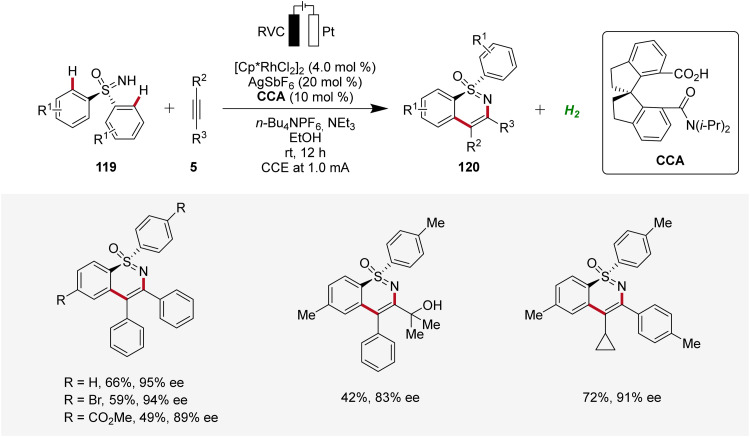
Enantioselective rhodaelectro-catalyzed C–H annulation of sulfoximines 119.

Very recently, Shi and Zhou applied the rhoda-electrocatalysis strategy^12^ to the C–H annulation of sulfoximines 119, where a chiral carboxylic acid (CCA) was effective in controlling enantioselectivity.^44^ The *S*-stereogenic products 120 were obtained in moderate to good yields and enantioselectivities (Scheme 36).^44^

## 3 3d metallaelectro-catalyzed alkyne annulations

### 3.1 Cobaltaelectro-catalyzed C–H activation

**Scheme 37:**
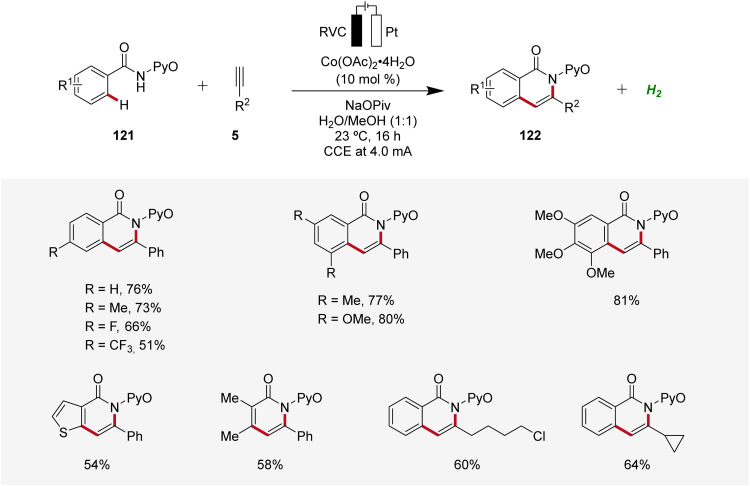
Cobaltaelectro-catalyzed C–H/N–H alkyne annulation with benzamides 121.

Cobalt, an economically viable and Earth-abundant transition metal, has emerged as one of the foremost contenders for facilitating carbon–carbon and carbon–heteroatom bond-forming reactions.^45^ In 2018, Ackermann reported on the first cobaltaelectro-catalyzed C–H/N–H alkyne annulation of benzamides 121 (Scheme 37).^46^ The synthesis of isoquinolinones 122 was achieved under exceedingly mild and environmentally-friendly conditions, employing an undivided cell equipped with a platinum plate cathode and a reticulated vitreous carbon (RVC) anode under galvanostatic electrolysis. In particular, the pyridine oxide directing group (PyO) proved to be suitable for facilitating the electrocatalytic C–H/N–H annulation. Under the optimized electrochemical conditions, a wide substrate scope was identified, demonstrating broad applicability. Thus, alkynes 5 having cyclopropyl, alkyl chloride, and ester functional groups were found to be viable substrates.^46^

**Scheme 38:**
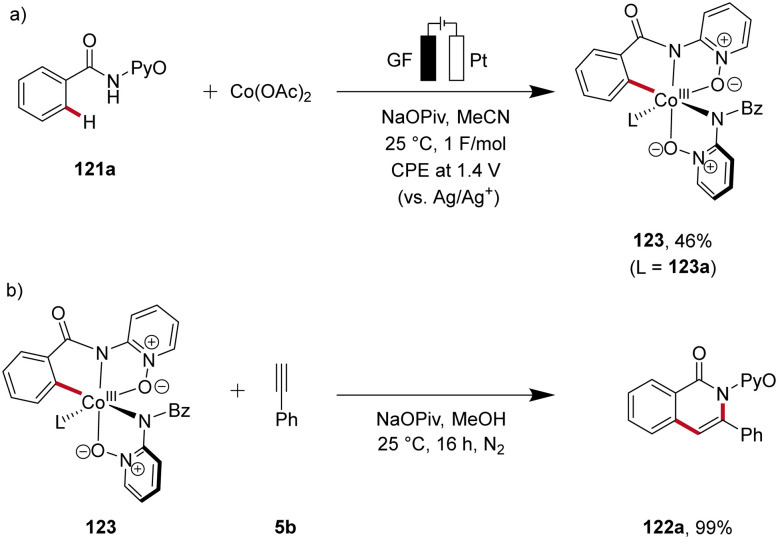
Key experiments to unveil the mechanism of the C–H/N–H alkyne annulation.

Recently, in 2020, Ackermann reported key studies that provided mechanistic insights into the mode of action of cobalta-electrocatalysis (Scheme 38).^47^ Herein, the electrosynthesis of the cyclometallated cobalt(iii) complex 123 was achieved, which was further confirmed to be a key intermediate in the electrocatalytic process (Scheme 38a). Thus, when this intermediate 123 is reacted with the alkyne 5b in the absence of electricity, the annulated product 122a is formed in 99% yield (Scheme 38b). This result verifies a facile reductive elimination from cobalt(iii) for the C–H/N–H annulation without the need for an oxidation to cobalt(iv), as found for the C–O bond forming pathway in C–H alkoxylation *via* oxidation-induced reductive elimination.^47^

**Scheme 39:**
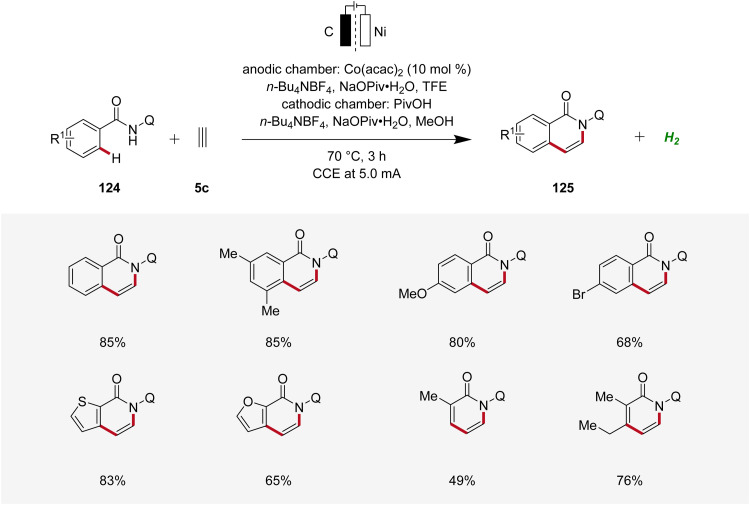
Cobaltaelectro-catalyzed C–H/N–H ethyne annulation.

Concurrently, Lei applied the cobaltaelectro-catalyzed C–H annulation strategy using 8-quinolinyl (Q) substituted benzamides 124 and ethyne 5c using a divided cell setup to yield isoquinolinones 125 (Scheme 39).^48^ This approach exhibited broad substrate scope, tolerating various benzamide and acrylamide derivatives 124 as suitable substrates.^48^

**Scheme 40:**
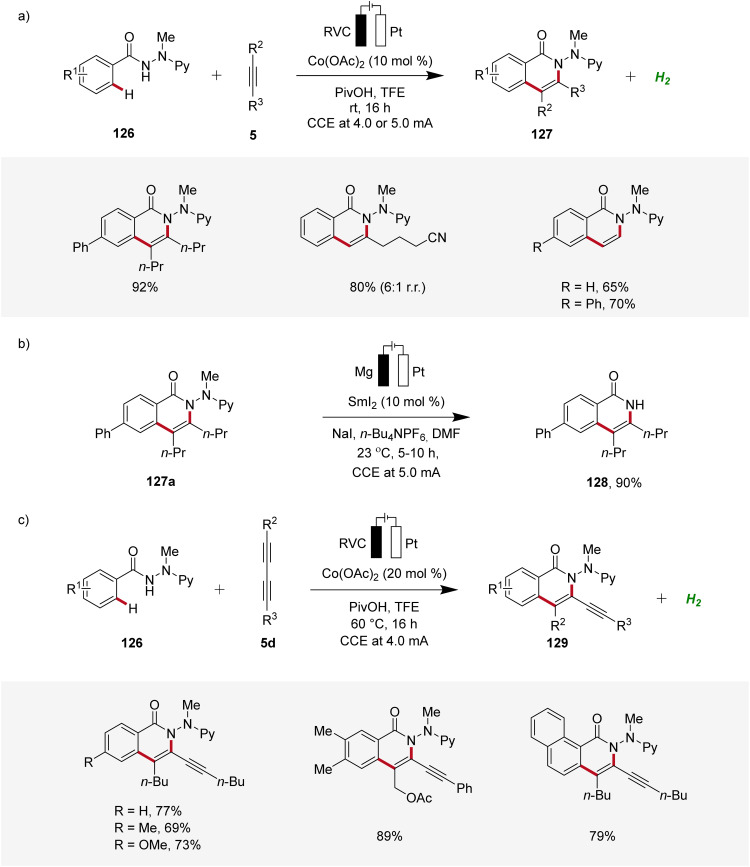
Cobaltaelectro-catalyzed C–H/N–H annulations with electro-removable hydrazides 126.

Furthermore, Ackermann demonstrated the applicability of cobaltaelectro-catalyzed C–H/N–H alkyne annulation to benzamide 126 bearing an electro-removable *N*-2-pyridylhydrazide auxiliary under exceedingly mild conditions at room temperature with ample scope (Scheme 40a).^49^ Interestingly, the auxiliary could be easily cleaved electro-reductive samarium-catalysis, exhibiting the utility of this strategy (Scheme 40b).^49^ In 2019, Ackermann further developed a cobaltaelectro-catalyzed C–H/N–H annulation approach, specifically targeting the challenging substrate class of 1,3-diynes 5d (Scheme 40c).^50^ The selectivity challenges associated with 1,3-diynes 5d are significantly more intricate compared to those observed with internal alkynes. Remarkably, the developed approach demonstrated excellent substrate scope and significant compatibility with various functional groups. Also here, the hydrazide directing group could be easily cleaved through samarium-electrocatalysis.^50^

**Scheme 41:**
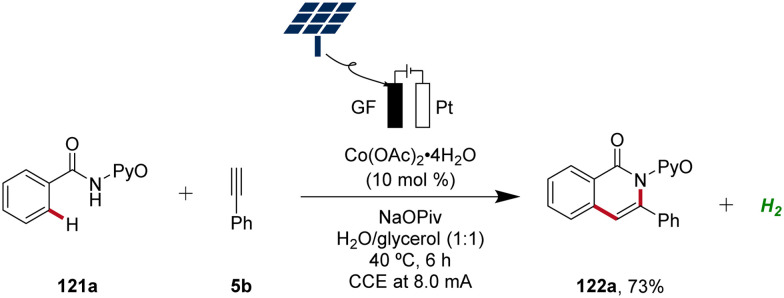
Cobaltaelectro-catalyzed C–H alkyne annulation in aqueous glycerol driven by natural sunlight.

In 2020, Ackermann further demonstrated the green aspects of cobaltaelectro-catalyzed C–H activation by performing the synthesis of isoquinolinones 122 in biomass-derived glycerol in an user-friendly undivided cell under galvanostatic electrolysis.^51^ Importantly, the direct use of renewable energy sources, including sunlight and wind power, to drive this sustainable and resource-economic electrocatalytic transformation was established, showcasing the robustness and practicality (Scheme 41).^51^

**Scheme 42:**
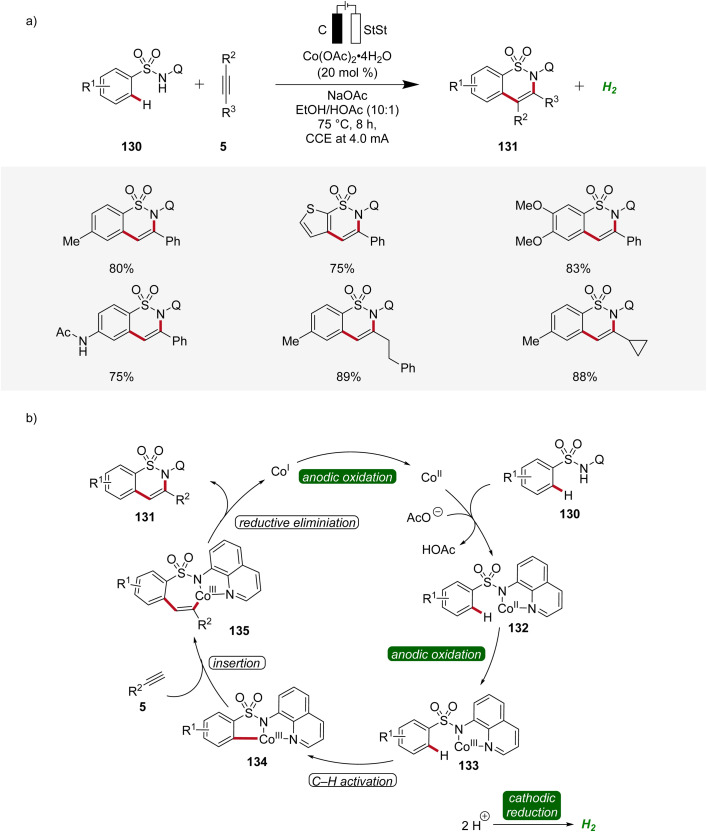
Versatility and schematic catalytic cycle for the synthesis of sultams 131*via* cobaltaelectro-catalyzed C–H annulation.

In 2020, Lei applied the cobalta-electrocatalysis strategy to synthesize structurally diverse sultams 131 by the annulation of sulfonamides 130 with alkynes 5 (Scheme 42).^52^ The reaction was performed in an undivided cell under galvanostatic electrolysis. Various sulfonamides 130 and alkynes 5 substituted with different functional groups were explored which delivered the broad substrate scope of this method. Internal alkynes 5 also produced the annulation products in moderate to high yields (Scheme 42a). Mechanistic studies revealed that first, the cobalt(ii) species is coordinated by the sulfonamide substrate 130 to produce the cobalt(ii) complex 132 which is oxidized to generate the cobalt(iii) intermediate 133. Next, the cyclometallated cobalt(iii) complex 134 is generated by C–H activation followed by insertion of alkyne 5. Lastly, reductive elimination leads to the final annulation product 131 (Scheme 42b).^52^

### 3.2 Cupraelectro-catalyzed C–H activation

**Scheme 43:**
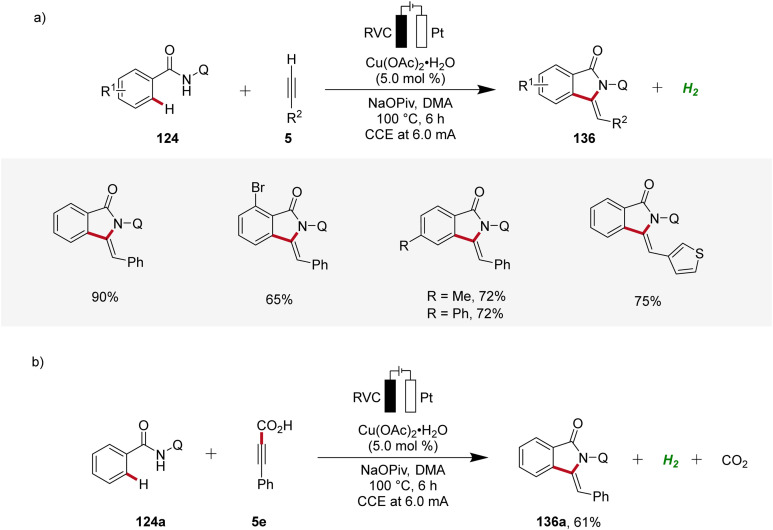
Cupraelectro-catalyzed C–H alkyne annulations to construct isoindolones 136.

Copper plays a significant role in transition metal-catalysis being an Earth-abundant and cost-effective transition metal with unique properties and versatility.^53^ In 2019, Ackermann reported on the first cupraelectro-catalyzed C–H activation (Scheme 43).^54^ This resource-economic strategy allowed for alkyne annulation with benzamides 124 and terminal alkynes 5 and exhibited excellent functional group tolerance (Scheme 43a). Interestingly, the cupra-electrocatalysis led to the formation of isoindolones 136, rather than isoquinolones as observed under cobalta-electrocatalysis.^46,49^ In addition, the strategy also allowed for decarboxylative C–H/C–C functionalizations by electrocatalysis (Scheme 43b).^54^

**Scheme 44:**
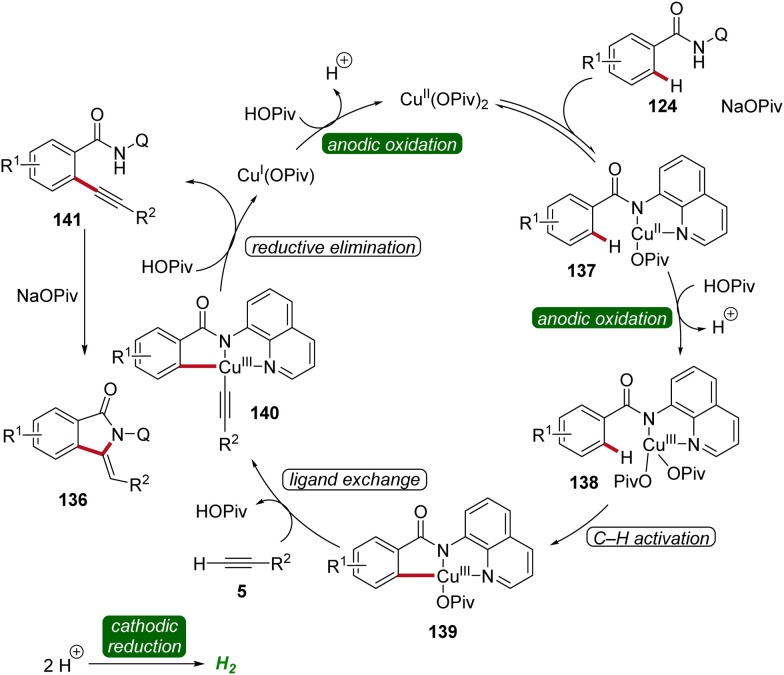
Catalytic cycle for the cupraelectro-catalyzed C–H activation leading to isoindolones 136.

Based on detailed mechanistic investigations, including H/D exchange experiments, kinetic isotope effect studies, and *in operando* kinetic analyses as well as cyclic voltammetry studies, a plausible mechanism was described (Scheme 44). Hence, by coordination of the substrate 124 and anodic oxidation, the formation of the copper(iii) intermediate 138 is promoted. This species then undergoes C–H activation to form the cupra(iii)-cycle 139, followed by metalation of the terminal alkyne 5. The subsequent reductive elimination delivers the C–H alkynylated arene 141, which undergoes cyclization to furnish the desired isoindolone product 136. The copper(i) complex is then oxidized at the anode to regenerate the catalytically active high-valent copper species (Scheme 44).^54^

### 3.3 Enantioselective 3d metallaelectro-catalyzed alkyne annulations

**Scheme 45:**
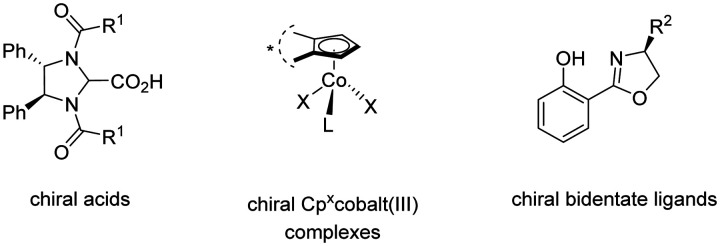
Control of enantioselectivity in high-valent cobalt-catalyzed C–H activation.

The 3d transition metal cobalt has recently emerged as a particularly promising catalyst for enantioselective C–H activation.^55^ Its low cost, abundant availability, and unique reactivity make it an attractive alternative to the earlier established 4d and 5d transition metals such as palladium, rhodium, and iridium. Hence, in the field of high-valent cobalt-catalyzed C–H activation, various strategies for controlling enantioselectivity have been identified (Scheme 45).^56^ In 2018, Ackermann introduced newly designed C_2_ symmetric chiral carboxylic acids to enable the first examples of enantioselective high-valent cobalt-catalyzed C–H activation.^57^ Later, in 2019, Cramer identified chiral cyclopentadienyl cobalt(iii) complexes as viable pre-catalysts for C–H activation reactions with high enantioselectivity.^58^ Subsequently, in 2022, Shi^59^ and Niu^60^ applied chiral salicyloxazoline ligands, first described by Bolm,^61^ for enantioselective cobalt-catalyzed C–H activations employing bidentate directing groups.

**Scheme 46:**
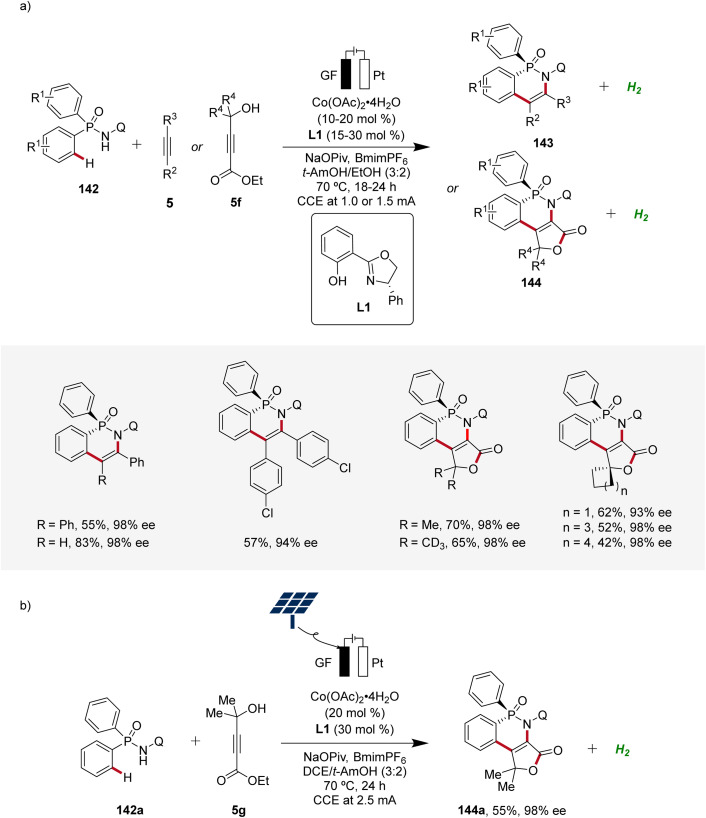
Enantioselective cobaltaelectro-catalyzed C–H alkyne annulations for the synthesis of *P*-stereogenic compounds 143 and 144.

In 2023, Ackermann delineated the first enantioselective cobaltaelectro-catalyzed C–H activations (Scheme 46).^62^ Employing L1 as ligand, the enantioselective C–H annulation of arylphosphinic amides 142 and alkynes 5 successfully yielded *P*-chiral cyclic phosphinic amides 143 with exceptional enantioselectivity and broad substrate scope (Scheme 46a). Furthermore, the efficacy of this transformation extends beyond conventional alkynes 5, as the cascade annulation involving alkynoates 5f was also accomplished. Importantly, it could be demonstrated that the enantioselective cobalta-electrocatalysis can directly be driven by natural sunlight as a renewable form of energy using a solar-panel (Scheme 46b).^62^

**Scheme 47:**
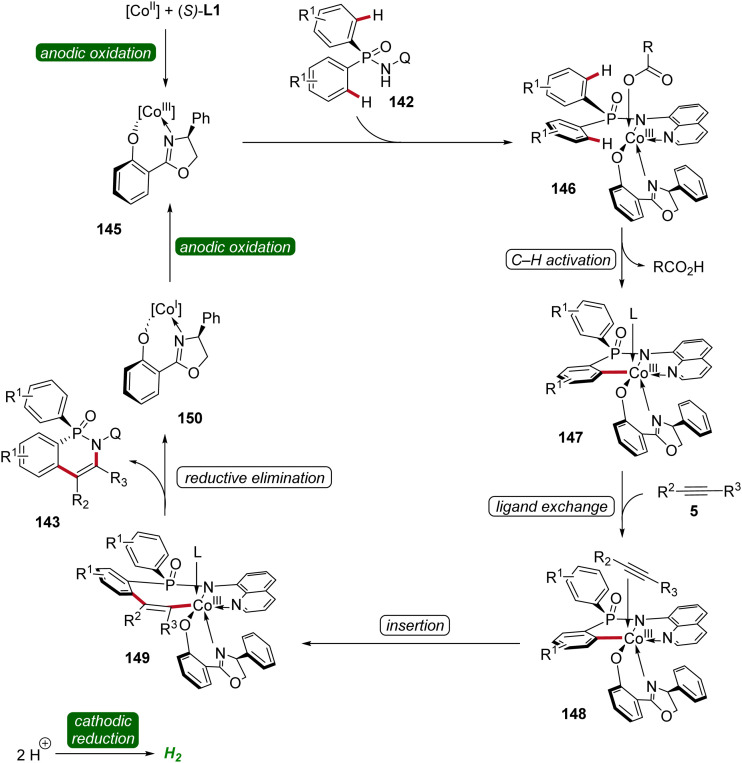
Schematic catalytic cycle for the cobaltaelectro-catalyzed enantioselective C–H annulation.

Based on mechanistic studies and previous reports,^47,59,62,63^ a reaction mechanism is depicted (Scheme 47). First, anodic oxidation of the cobalt(ii) pre-catalyst generates the active chiral cobalt(iii), which is coordinated by the chiral ligand L1 und substrate 142 to form intermediate 146. Next, the cyclometallated cobalt(iii) intermediate 147 is formed *via* enantioselective C–H activation, followed by coordination and migratory insertion of alkyne 5. Subsequent reductive elimination delivers the chiral compound 143 along with cobalt(i) complex 150. Finally, 150 is re-oxidized by anodic oxidation to complete the catalytic cycle.

**Scheme 48:**
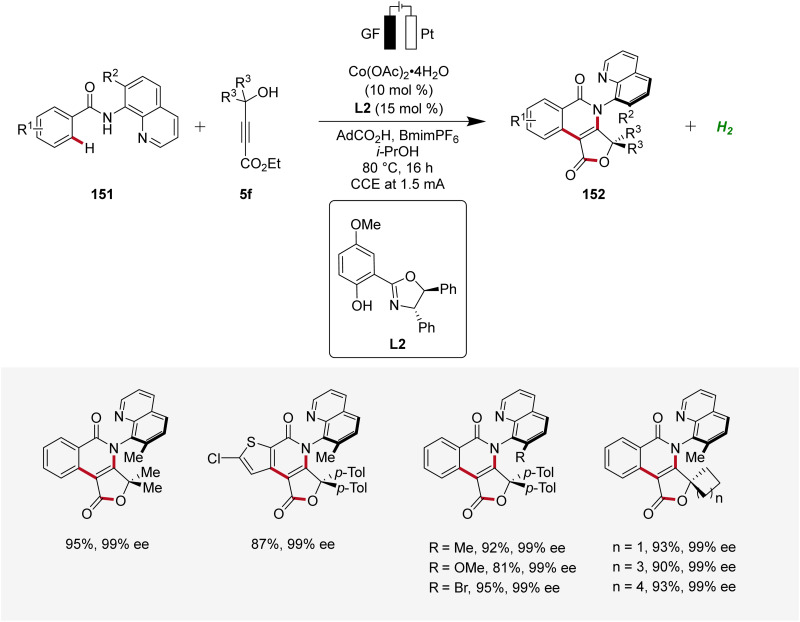
Atroposelective cobaltaelectro-catalyzed C–H alkyne cascade annulation.

Moreover, Ackermann devised the first enantioselective cobaltaelectro-catalyzed synthesis of axially chiral compounds 152 (Scheme 48).^62^ The atropo-chiral products 152 were accessed with excellent yields and enantiomeric purities. Notably, the atroposelective cobalta-electrocatalysis proved to be scalable using cost-effective stainless steel as cathode material instead of the commonly used precious platinum.^62^

**Scheme 49:**
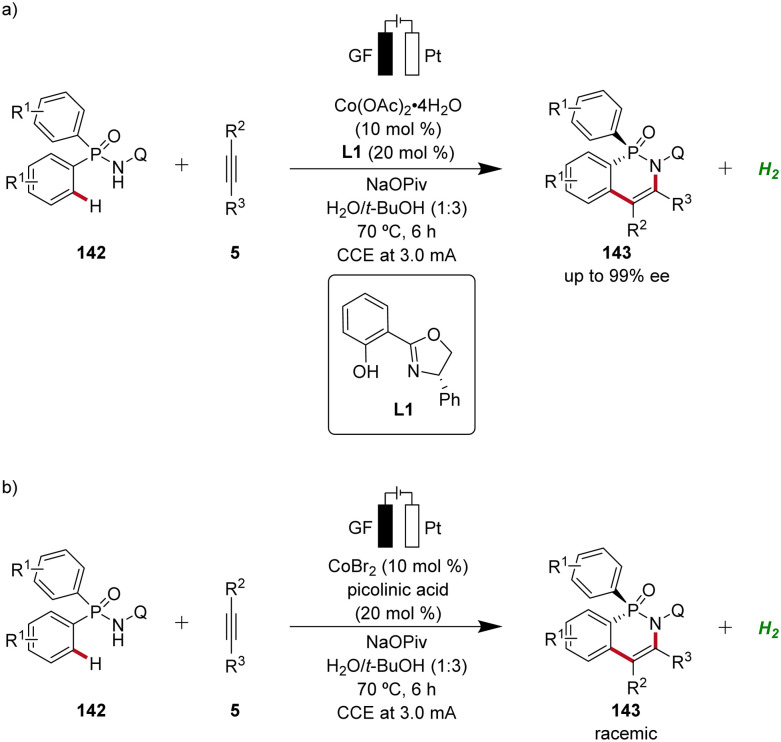
Enantioselective and non-enantioselective cobaltaelectro-catalyzed C–H annulation for the synthesis of *P*-stereogenic phosphinic amides 143.

Following the pioneering studies by Ackermann,^62,63*a*^ enantioselective cobaltaelectro-catalyzed reactions have flourished with several reports.^56^ Ling applied the cobalta-electrocatalysis strategy for the enantioselective^63*b*^ and non-enantioselective^64^ synthesis of *P*-stereogenic compounds 143 using an aqueous solvent system (Scheme 49).^63*b*,64^

**Scheme 50:**
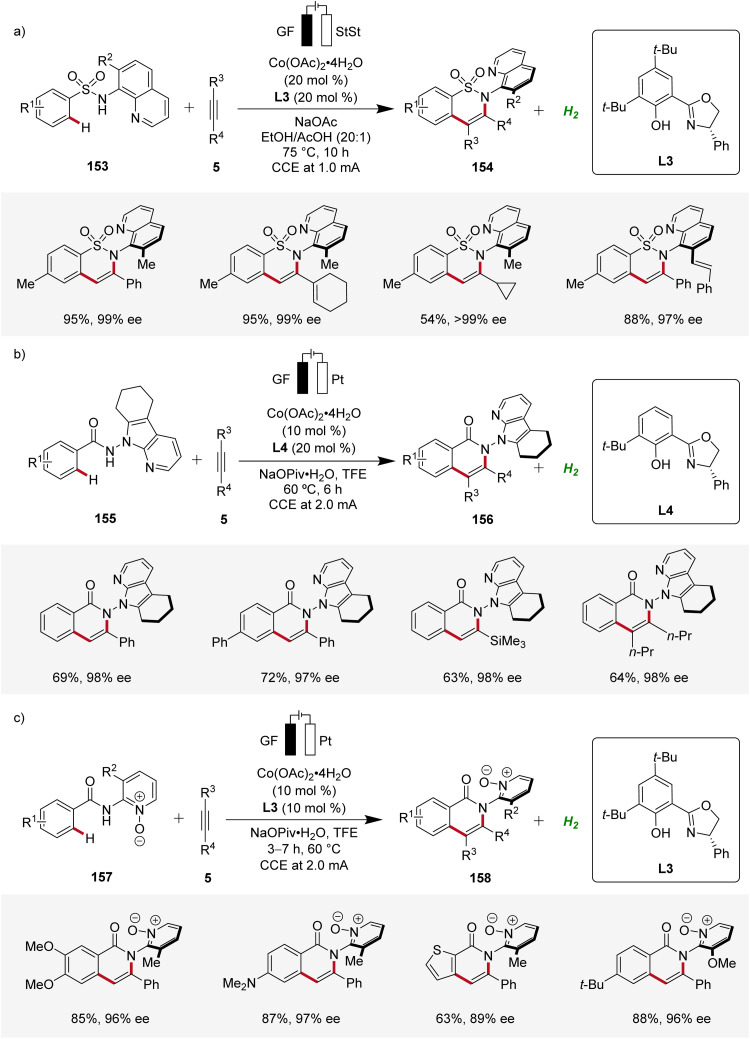
Atroposelective cobaltaelectro-catalyzed C–H annulations with diverse directing groups.

Furthermore, Niu contributed significantly demonstrating several cobaltaelectro-catalyzed annulation reactions with alkynes for the assembly of axially chiral molecules using chiral salox-based ligands (Scheme 50). First, an atroposelective annulation of alkynes 5 with sulfonamides 153 was reported, forming atropo-chiral sultams 154 with high level of selectivity (Scheme 50a). The strategy consisted a broad scope and high enantioselectivity in the products.^65^ Later, Niu devised an atroposelective annulation reaction with internal and terminal alkynes 5, where 7-azaindole derived directing groups were employed, leading to versatile *N*–*N* axially chiral compounds 156 with high levels of enantioselectivity (Scheme 50b).^66^ In addition, Niu reported an atroposelective annulation of alkynes 5 employing benzamides 157 bearing pyridine-*N*-oxide derived directing groups (Scheme 50c).^67^

## 4 Conclusion

The intersection of organic synthesis, renewable energy, and hydrogen economy through metallaelectro-catalysis reveals seminal opportunities towards sustainable development. Thus, electrocatalytic processes, powered by renewable forms of energy, can provide an alternative to traditional chemical methods, reducing the need for harsh reagents and minimizing waste. Importantly, pairing of organic synthesis to the valuable hydrogen evolution reaction (HER) enables a prospective integration into a decentralized green hydrogen economy.

Metalla-electrocatalysis, a rapidly evolving field, has emerged as a cutting-edge technique to forge new synthesis routes. Given that past research on C–H annulation reactions has primarily focused on precious transition metals, such as rhodium and ruthenium, it is anticipated that future efforts will increasingly emphasize more the Earth-abundant and less toxic transition metals. Hence, the commencing exploration of 3d transition metals, such as cobalt and copper, has paved the way for the development of resource-economical and environmentally benign processes. Moreover, the ability to control enantioselectivity, which is an essential feature in the synthesis of pharmaceuticals and agrochemicals, has very recently been accomplished and offers novel opportunities towards full selectivity control.

As electrocatalysis continues to advance, it is expected that this innovative technique will become an integral part of the toolkit of organic chemists. Its ability to redesign organic synthesis, coupled with its potential to be integrated into a decentralized green hydrogen economy, bodes well for a future in which electrocatalysis plays a central role in advancing sustainable chemical processes.

## Data availability

No primary research results have been included and no new data were generated or analyzed as part of this review.

## Conflicts of interest

There are no conflicts to declare.
